# Natalizumab stabilizes physical, cognitive, MRI, and OCT markers of disease activity: A prospective, non-randomized pilot study

**DOI:** 10.1371/journal.pone.0173299

**Published:** 2017-04-20

**Authors:** Garrick D. Talmage, Oscar J. M. Coppes, Adil Javed, Jacqueline Bernard

**Affiliations:** 1 Department of Ear, Nose and Throat, University of Colorado, Aurora, CO, United States of America; 2 Department of Neurology, Oregon Health & Science University, Portland, Oregon, United States of America; University of New Mexico, UNITED STATES

## Abstract

Natalizumab is an effective therapy for multiple sclerosis (MS). Its effectiveness has been demonstrated in several clinical and imaging studies. The objective of this study was to further demonstrate the efficacy of natalizumab using a comprehensive battery of clinical and imaging markers in the same cohort of patients followed longitudinally, hence capturing the multi-faceted nature of the MS disease process. A prospective, open-label, pilot study of 20 MS patients treated with natalizumab was conducted. High resolution MRI, Symbol-Digit Modalities Test (SDMT), and Optical Coherence Tomography (OCT) scans were obtained at baseline, 48, and 96 weeks. 15 patients completed the study. Natalizumab treatment decreased Expanded Disability Status Scale score (EDSS) and no change in SDMT, Brain Parenchymal Fraction (BPF), or any of the OCT markers of retinal degeneration was observed. Thalamic and whole brain volume as assessed by Percentage Brain Volume Change (PBVC) showed continuous deterioration. Higher baseline T2 lesion load correlated with increased rate of PBVC at 96-weeks (r = 0.566, R^2^ = 0.320, p = 0.035) and thalamic volume loss (r = -0.586, R^2^ = 0.344, p = 0.027). Most patients, 93%, achieved no evidence of disease activity (NEDA) at 2 years, likely due to early disease duration and lower initial baseline lesion load. This study further demonstrates stabilization of clinical and imaging markers of disease activity during natalizumab treatment.

## Introduction

Relapsing-remitting multiple sclerosis (RRMS) is an inflammatory and neurodegenerative disease of the central nervous system (CNS) resulting in progressive neuronal and axonal loss in the gray and white matter, leading to both physical and cognitive disability [1]. Cognitive dysfunction occurs early in the disease course of MS and is an important factor in the quality of life of patients [2–4]. Physical and cognitive decline in RRMS has been correlated to changes in several imaging modalities [5–7]. These studies have hypothesized that irreversible neurodegeneration may occur early in the disease course and may be central to the development of long-term physical and cognitive disability.

Treatments that prevent neurodegeneration and axonal loss may be best suited to prevent long-term disability in MS. Natalizumab (Tysabri, Biogen Idec/Elan) is a recombinant monoclonal antibody against the α_4_-subunit of α_4_β_1_-integrin expressed on leukocytes [8,9]. By preventing migration of leukocytes across the blood-brain barrier, natalizumab and has been shown to limit lesion formation and reduce axonal loss [9,10]. Several studies have shown that natalizumab is effective in reducing markers of physical disability (EDSS), cognitive decline (SDMT), and various MRI markers of disease activity (Gadolinium (Gd)-enhancing lesions, T2 lesions, brain atrophy) [11–13]. There is little if any information regarding the effects of natalizumab on the OCT surrogate marker of disease activity in MS. More recently, the effectiveness of MS treatments have been gauged by using another composite metric known as no evidence of disease activity (NEDA), which usually includes no progression on EDSS scores, lack of any new MRI activity (new contrast enhancing lesions and new or enlarging T2 lesions), and lack of any relapses [14,15]. This 3-component metric is referred to as NEDA-3. Other clinical or MRI measures can be added to NEDA-3, more common one being brain atrophy due to its moderate correlation with long-term disability, formulating NEDA-4. It is hypothesized that MS treatments that are associated with a higher NEDA score in the earlier part of the disease course may be better at reducing long-term disability in MS patients.

Until recently, use of natalizumab as a first-line treatment in RRMS has been limited due to concerns regarding risk of Progressive Multifocal Encephalopathy (PML) [16]. PML is an aggressive demyelinating disease caused by the reactivation of John Cunningham Virus (JCV) and a subsequent CNS infection. Highly sensitive serum JCV testing is available and accumulating data suggests that patients who are seronegative or have low titers have reduced risk of developing PML [17]. Hence, natalizumab is increasingly used as a first-line disease modifying treatment (DMT) for patients stratified to a lower risk category [18,19].

Although several studies have examined the effects of Natalizumab on select imaging and clinical markers of disease activity in MS, there has not been a study that has employed a comprehensive battery of clinical and imaging measures in the same cohort of patients longitudinally to examine treatment effect on multiple markers of disease progression. The objective of this prospective, non-randomized, pilot study was to assess the efficacy of Natalizumab in RRMS patients using several metrics of physical, cognitive, and imaging markers of disease activity, such as EDSS, SDMT, brain volume, thalamic volume, OCT, and NEDA-3 composite score. We hypothesized that natalizumab treatment will result in stabilization or improvement in these measures and will be associated with a high NEDA-3 score.

## Methods

### Patients

Twenty patients were enrolled prospectively into a 96-week, open-label, single center study. All patients gave written informed consent.

The trial was registered with ClinicalTrails.gov using the identifier: NCT01071512; information can be found at https://clinicaltrials.gov/ct2/show/NCT01071512.

Eligible patients were males and females age 18–60 inclusive; had a diagnosis of RRMS according to the 2005 revised McDonald criteria [20]; had an Expanded Disability Status Scale (EDSS) score of 0.0 to 7.0 inclusive [21]; had disease activity defined by either 1 documented relapse during the previous year, 2 documented relapses during the previous 2 years, or one or more new lesions on MRI (Gd-enhancing or T2 hyperintense) during a screening MRI; were neurologically stable with no evidence of relapse or corticosteroid treatment within 30 days prior to treatment; and were naïve to natalizumab. Those who were ineligible to receive natalizumab due to immunosuppression, immune deficiency, or malignancy were excluded. All patients in the study received 300 mg of natalizumab intravenously every 4 weeks [22]. Every 24 weeks, patients were seen by the treating neurologist for a physical exam and to monitor for relapses and adverse events. Blood and urine laboratory tests were also performed, including an anti-natalizumab antibody test at 24 weeks. If needed, patients saw the treating neurologist for new or worsening symptoms and were treated with intravenous methylprednisolone for relapses at the discretion of the treating neurologist. In addition to follow-up physical exams, measurements evaluating neurodegeneration and cognitive function were obtained according to the schedules described in the sections below.

The study was conducted at the University of Chicago Medical Center (UCMC) and approved by the Institutional Review Board of the University of Chicago, IL, USA under protocol number 10-094-A. Patients at UCMC that were initiating treatment with natalizumab and interested in participating were referred for eligibility.

### Demographic, clinical, and imaging characteristics at baseline

**Fig 1:**
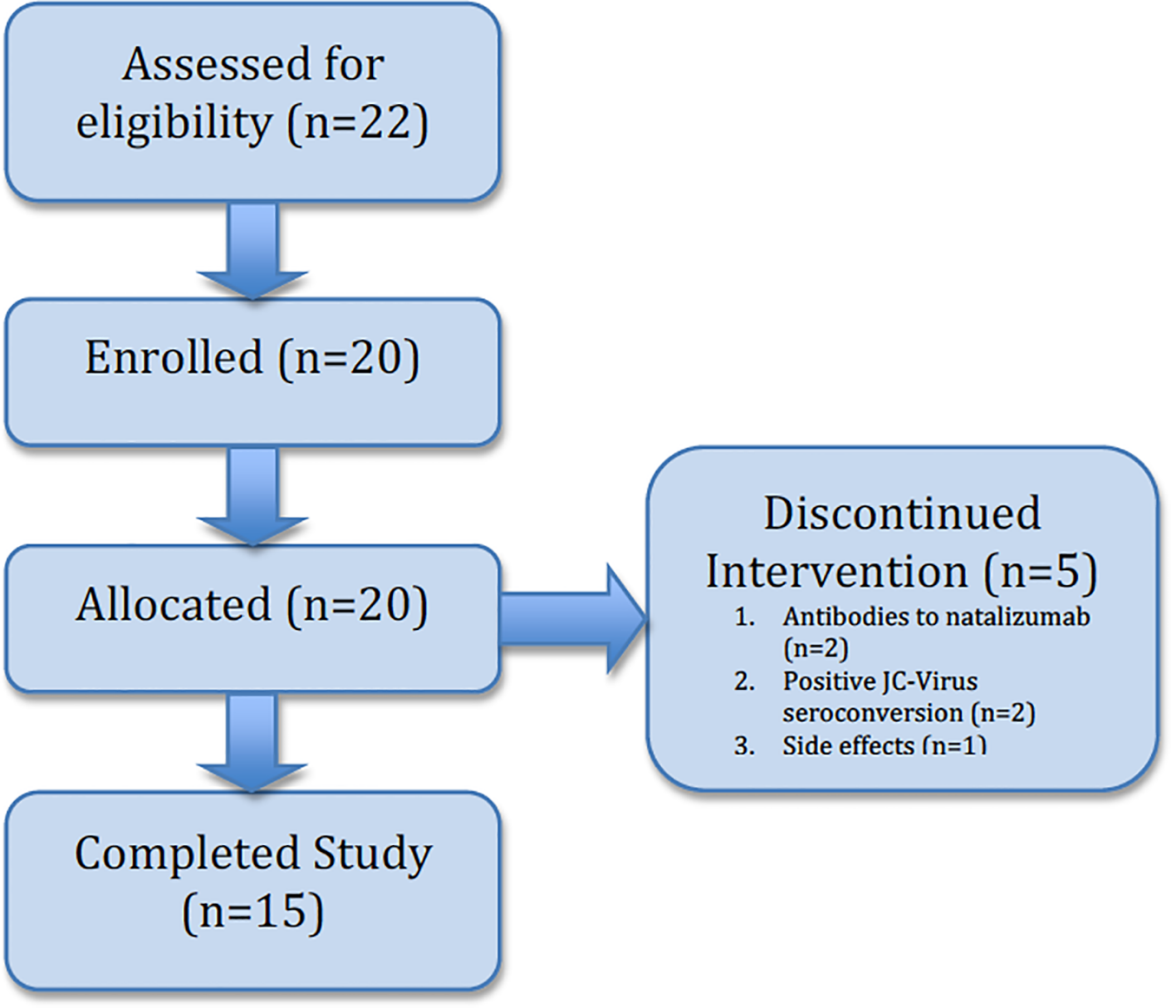
TREND flow diagram describing the number of patients the trial screened, enrolled, allocated, completed and discontinued the intervention.

Twenty-two patients were screened for the study, and twenty were actually enrolled **(Fig 1)** between 2010 and 2013. Five patients withdrew from the study while on treatment: one patient due to side effects of natalizumab; two patients due to development of antibodies to natalizumab; and two patients after converting to a positive anti-JC Virus antibody test. One patient’s MRI data at year one and year two was unable to be included due to severe distortion; however, this patient provided the clinical and OCT data for the study. Baseline demographic data for those patients completing the study are included in **Table 1**. SDMT scores were converted to z-scores using published age and education based norms [23,24]. Disease duration was defined as time from RRMS diagnosis to the start date of natalizumab treatment.

**Table 1:** Baseline characteristics of study cohort.

Characteristic*		Patients completing 96 weeks of treatment
No. of patients		15
No. of women (%)		13 (87)
Age (years)		39 ± 9
Race (%)	Caucasian	8 (53)
	African-American	6 (40)
	Other	1 (7)
BMI		30 ± 9
Years of Education > 12 (%)		13 (87)
Previous use of DMT (n, %):	0 DMT	4 (27)
	1 DMT	5 (33)
	≥2 DMT	6 (0.4)
Disease Duration (years)	Median ± SD	3 ± 4.6
	Range	0–14
	Disease Duration > 2 years (%)	9 (60)
MedianT2 Lesion Load (cm^3^)		1.54 ± 9

* values reported are mean ± SD unless otherwise noted.

BMI–Body Mass Index; DMT–Disease Modifying Therapy.

### MRI collection and analysis

The MR scans were obtained on a 3T Phillips scanner (Philips Medical Systems, Best, The Netherlands) at the University of Chicago. Scans were obtained at baseline, 48 weeks, and 96 weeks, and all images were reviewed by a neuroradiologist to evaluate for disease progression and to assess for any findings not consistent with MS. Imaging parameters used were: 3D T1-weighted Turbo Field Echo (3DT1TFE) TR = 8 ms, TE = 3.6 ms, flip angle = 15°, voxel size = 1 x 1 x 1 mm^3^ and FLAIR images TR = 11,000 ms, TE = 125 ms, TI = 2800 ms, both with matrix size = 256 x 256, FOV = 224 x 224 mm.

Percentage brain volume change (PBVC) was estimated using SIENA [25,26]. Subcortical segmentation of each scan was performed using FIRST [27]. Volumes of thalamus and hippocampus were multiplied by the normalized brain parenchymal fraction (BPF) obtained through SIENAX to adjust for head size. For gray and white matter segmentation, white matter lesions were manually assigned intensities matching the surrounding normal appearing white matter using FreeView [28]. T2 lesion load was determined by using JIM (v.7, Xinapse System) [29].

### SD-OCT collection and analysis

Spectral domain OCT (SD-OCT) scans were performed using a Heidelberg Spectralis OCT (Heidelberg Engineering, Inc, Heidelberg, Germany) by a trained technician. Scans were performed without pupil dilation. Patients were scanned in both eyes at baseline, 24 weeks, 48 weeks, 72 weeks, and 96 weeks. High-quality images had signal strength of approximately 26 dB, uniform brightness, and crisp borders of blood vessels. Scanning protocol included a circular 6mm scan centered on the optic nerve head and a volumetric scan of the macula centered on the fovea (73 horizontal B-scans covering superior-to-inferior distance of 6mm, 25 frames per eye, 20° x 20° scans, and an automatic real-time mean value set at 9). Global and temporal region Retinal Nerve Fiber Layer (RNFL) thickness, total macular volume (MV), and central foveal thickness (CFT) of each eye were recorded.

### Tests of cognitive function and neurologic impairment

Cognitive function was assessed by the SDMT. SDMT measures cognitive processing by having subjects match numbers with target symbols, with the symbol-number legend printed at the top of the test sheet. The same form of the SDMT was used for each assessment, which occurred at baseline, 48 weeks, and 96 weeks. At each assessment, subjects were given two forms of the test. First, subjects responded by writing the number associated with the symbol. Afterwards, subjects verbalized the number associated with the symbol. The oral version was used as the outcome measure by recording the number of correct responses made in 90 seconds. Neurologic impairment was quantified using EDSS by an investigating physician. EDSS scores were recorded at baseline, 24 weeks, 48 weeks, 72 weeks, and 92 weeks.

### Statistical analysis

Statistical analysis was performed using SPSS 23.0 (IBM Corp., Armonk, NY). Student’s t-test and Pearson’s correlations were used as appropriate to analyze continuous independent variables. Mixed effects models were used to model longitudinal data, using either an unstructured, compound symmetry, or autoregressive covariance structure [30]. As this was a pilot and hypothesis generating study, a Bonferroni correction was not applied and probabilities less than 0.05 were considered to be statistically significant.

## Results

### Change over time in clinical progression and imaging metrics of neurodegeneration

Mixed models were employed to determine if clinical and imaging metrics changed over time, using time as a fixed effect. Each model was first fit to an unstructured covariance matrix and then switched to a compound symmetry or autoregressive matrix if– 2 likelihood ratios demonstrated them not to be inferior. Longitudinal raw data in **Table 2**represents only patients that completed all 96 weeks of treatment (n = 15); however, the mixed models incorporated those patients who dropped out of the study early. Reported p-values are for Type III tests of the fixed effect of time. As an ordinal variable, EDSS was instead fit to a generalized estimating equations model.

**Table 2:** Longitudinal clinical and imaging metrics over 96-week treatment period.

Metric*	Baseline	Week 24	Week 48	Week 24	Week 96	p-value
Oral SDMT z-score	-1.5 ± 0.9	-	-1.2 ± 1.0	-	-1.2 ± 0.9	0.17
EDSS, median (IQR)	3.0 (2.5–4.0)	2.0 (2.0–3.5)	2.5 (2.0–3.5)	2.5 (2.0–3.13)	2.5 (2.0–3.0)	**0.007**
Brain Parenchymal Fraction	0.970 ± 0.011	-	0.969 ± 0.012	-	0.971 ± 0.014	0.3
Gray Matter Fraction	0.521 ± 0.014	-	0.522 ± 0.008	-	0.520 ± 0.013	0.4
White Matter Fraction	0.479 ± 0.014	-	0.478 ± 0.008	-	0.480 ± 0.013	0.4
Normalized Thalamic Volume (mL)	13.9 ± 1.9	-	13.7 ± 2.0	-	13.7 ± 2.1	**0.02**
Normalized Hippocampal Volume (mL)	6.8 ± 0.8	-	6.7 ± 1.0	-	6.6 ± 0.9	0.9
Retinal Nerve Fiber Layer thickness (μM)	86 ± 13	85 ± 12	85 ± 13	85 ± 13	85 ± 13	0.6
Macular Volume (mm^3^)	8.4 ± 0.4	8.4 ± 0.4	8.3 ± 0.5	8.4 ± 0.4	8.4 ± 0.4	0.5

* values reported are mean ± SD unless otherwise noted.

SDMT–Symbol Digit Modalities Test; EDSS–Expanded Disability Status Scale; IQR–Interquartile Range.

Treatment with natalizumab resulted in a significant decrease in EDSS over the course of the treatment period (Table 2). The SDMT scores remained stable over time. There was no change in brain volume (BPF), gray or white matter fraction, and hippocampal volume. There was a small but significant decrease in the thalamic over the 96-treatment period. Also, PBVC showed a -0.8 ± 0.7% change at 48 weeks (p < 0.001) and a -1.2 ± 0.9% change at 96 weeks (p < 0.001). The OCT markers of neuronal integrity (RNFL and macular volume) also remained stable over 96 weeks. In terms of NEDA-3, 93% of patients achieved this composite measure.

### Baseline T2 lesion volumes correlate with change in thalamic volume and PBVC

**Fig 2:**
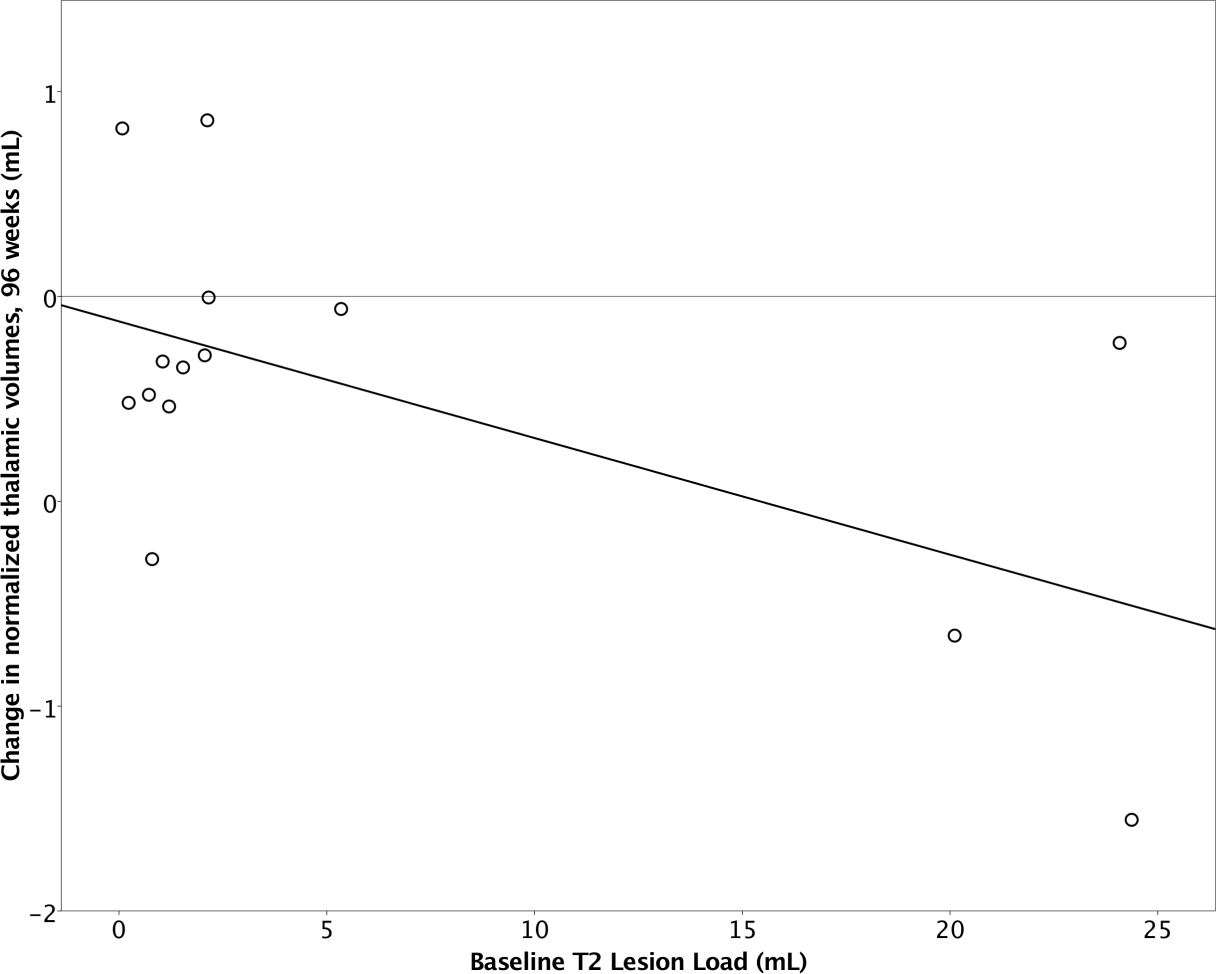
Higher baseline T2 lesion volume correlates with thalamic volume loss over 96 weeks.

Since there was continuous loss of thalamic and whole brain volume (PBVC) over 96 weeks, these measures were correlated with baseline level of disease activity as well as disease duration and age. Pearson’s correlation showed that higher baseline T2 lesion volumes were correlated with larger decreases in thalamic volume (r = -0.586, R^2^ = 0.344, p = 0.027), while disease duration and age showed no correlation with change in thalamic volume **(Fig 2)**. Higher baseline T2 lesion volumes also correlated with decrease in percentage brain volume change (PBVC) (r = 0.566, R^2^ = 0.320, p = 0.035).

### Change over time in cognitive function

**Fig 3:**
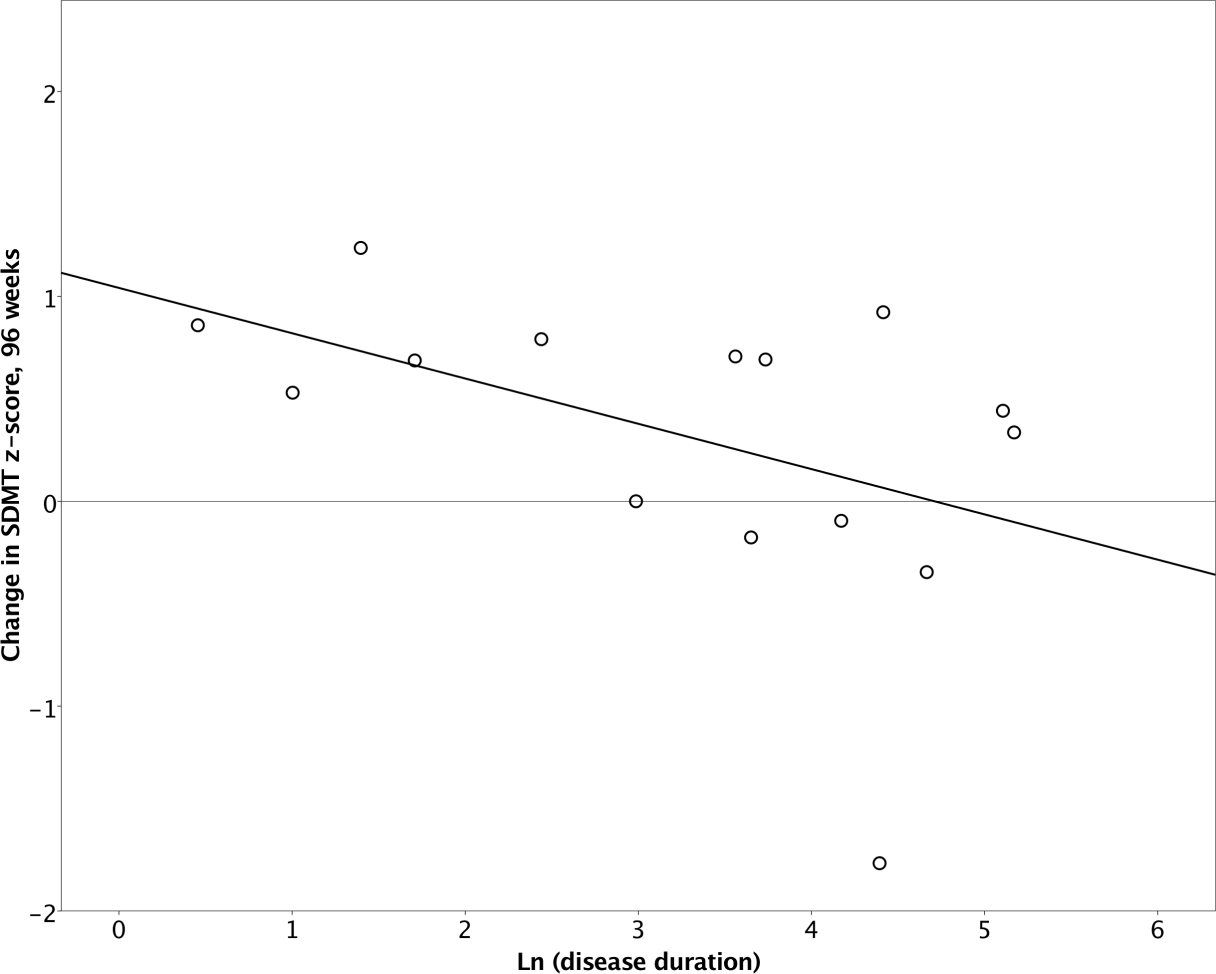
Correlation of disease duration with SDMT z-scores over the 96-week treatment period.

During natalizumab treatment over time, cognitive function remained stable. Age, baseline T2 lesion volume, baseline thalamic and hippocampal volume did not correlate with change in SDMT z-scores over 96-week period. For disease duration, the Shapiro-Wilk test rejected the hypothesis that the disease duration was normally distributed for the cohort (p = 0.002). A Q-Q plot demonstrated that the distribution was left-skewed and appeared logarithmic, so a natural logarithm was applied to disease duration for the remainder of the analysis. The distribution of ln(disease duration) was normal (Shapiro-Wilk, p = 0.14). At baseline, there was no correlation between natural logarithm of disease duration and SDMT z-scores (p = 0.8). Also, longitudinal changes in SDMT z-scores over 96-week period did not correlate with disease duration (B = -0.221, r = -0.460, p = 0.085), although a trend may be seen at lower disease duration correlating with a higher SDMT scores (**Fig 3)**.

To further confirm the relationship between disease duration and cognitive function, a mixed effects repeated measures model was created to model longitudinal changes in SDMT z-scores. Treatment time, natural logarithm of disease duration, and their interaction were defined as fixed effects. Based on -2 restricted likelihood criteria, a compound symmetry covariance matrix was not inferior to an unstructured matrix and thus was employed (F = 2.471, df = 4, p = 0.65). Type III tests of mixed effects showed that the effects of treatment time (p = 0.045) and the interaction of time and natural logarithm of disease duration (p = 0.036) were significant but not the natural logarithm of disease duration separately. Parameter effect sizes demonstrated that as compared to the 96-week time point, SDMT z-scores were lower at baseline (B = -0.95, p = 0.039) and 48-weeks (B = -1.09, p = 0.02), further indicating stabilization if not a mild improvement in cognitive function over the treatment period. However, disease duration alone did not influence cognitive function over time.

### Relationship between cognitive function and MRI markers of neurodegeneration

To evaluate MRI predictors of SDMT z-scores, exploratory correlations were performed between baseline SDMT z-scores and several variables: thalamic volume, hippocampal volume, brain volume (BPF), gray matter volume, T2 lesion load, and global and temporal RNFL thickness as measured by OCT. Higher SDMT z-scores were correlated to higher adjusted thalamic volume (r = 0.577, p = 0.008) and higher adjusted hippocampal volume (r = 0.621, p = 0.003) by Pearson’s correlation. There was no correlation between SDMT z-scores and gray matter volume, peripheral gray matter volume, BPF, T2 lesion volume or any of the OCT measures.

## Discussion

This prospective, open-label, observational study showed that natalizumab treatment over a 96-week period was associated with disease stability as measured by EDSS and SDMT and as per MRI surrogate markers of disease activity. OCT markers, including RNFL thickness and macular volume, did not change over time and remained stable for all patients. In 66% of patients, the EDSS actually improved by at least 0.5. A high percentage of patients, 93%, achieved NEDA-3. This high percentage of patients reaching NEDA-3 may be due to a greater proportion of subjects in this study having early disease (median disease duration 3 years) and low MRI disease burden (median T2 lesion volume 1.54). Previous observational studies have also reported a high percentage of patients achieving NEDA-3 with natalizumab [31], and taken together these observations resonant the concept of initiating effective treatments early in the MS disease course to prevent future disability. Patients with lower T2 lesion load had reduced thalamic atrophy and global atrophy as measured by PBVC. Baseline cognitive function was positively correlated with hippocampal and thalamic volumes. This short-term study using a comprehensive battery of clinical, MRI, and OCT measures showed that natalizumab is an effective therapy across multiple aspects of MS disease. It remains to be investigated whether long-term treatment with natalizumab over 10 years continues to be this effective in a large cohort of patients or perhaps delay the secondary progression of MS disease course.

Despite the effectiveness of natalizumab on most measures of disease activity in this study, thalamic volume and whole brain volume as assessed by PBVC continued to deteriorate. This is congruent with previous studies that have shown continued thalamic and brain volume loss despite the use of natalizumab over 2–3 years [13, 32–33]. The results herein show that despite the volume loss, the cognitive function as assessed by SDMT remained stable, at least in the short-term analysis. It remains to be investigated whether cognitive function starts to declines along with various indices of brain volume loss over a longer period of time. Brain volume loss is moderately correlated with long-term physical disability [34] and may very well be a good marker for cognitive disability as well, which remains to be investigated.

With the introduction and increased usage of natalizumab as a first-line drug for patients with RRMS who are JCV negative, questions remain about which patients should be selected for first-line treatment with natalizumab. This study suggests that patients with higher baseline T2 lesion load had greater loss of thalamic and whole brain volume. Previous studies have also suggested a positive correlation between lesion load and volume loss [35,36]. Hence, prevention of lesion formation appears to be important in preserving brain volume and perhaps long-term disability. In the future, a long-term treatment study of patients with newly diagnosed MS could be used to provide further evidence for neuroprotection as assessed by both cognitive function stabilization and improvement or prevention of brain volume loss.

Consistent with previous reports, baseline cognitive function closely correlated with subcortical structure such as the hippocampus and thalamus [6, 37]. Herein, higher SDMT z-scores were correlated with higher thalamic and hippocampus volumes. This effect appears to be independent of whole brain atrophy based on regression models. Atrophy and damage to white matter tracts associated with the hippocampus and thalamus likely results in deficits in processing and memory functions necessary for cognitive functions. These deficits may present early in the disease, and have been described in patients with Clinically Isolated Syndrome (CIS), the initial presentation of MS [37]. The natural history of cognitive decline in RRMS would be most clearly elucidated by using cognitive testing such as the SDMT in a future randomized placebo-controlled trial.

This study has several limitations. It is a small, observational study. However, our results are congruent with previous findings correlating higher thalamic and brain volumes with better cognitive function [6, 38]. The results of this study add to the previous findings and suggest that when natalizumab is used early in the disease course of MS, it may delay not only physical but also cognitive disability over time. Also, a higher than expected percentage of patients did not complete the study, but not due to efficacy reasons. Two patients dropped out due to JCV seroconversion and concerns about PML despite low risk in these patients. Although SDMT is a well-validated and repeatable estimate of cognitive function, it does not capture all aspects of cognitive dysfunction in MS patients.

This study, taken in the context of the effectiveness of natalizumab across several measures of disease activity, supports the use of natalizumab as a first-line drug, an area of increasing significance to both patients and physicians alike and furthering the concept of a higher level of no evidence of disease activity (NEDA) in MS.

## Supporting information

S1 File TREND checklist. (PDF)

S2 File Clinical Trial study protocol. (DOCX)

## References

[1] Compston A, Coles A. Multiple sclerosis. Lancet. 2008; 372: 1502–1517. doi: 10.1016/S0140-6736(08)61620-7 18970977 10.1016/S0140-6736(08)61620-7

[2] Rao SM, Leo GJ, Bernardin L, Unverzagt F. Cognitive dysfunction in multiple sclerosis. I. Frequency, patterns, and prediction. Neurology. 1991; 41: 685–691. 2027484 10.1212/wnl.41.5.685

[3] Rao SM, Leo GJ, Ellington L, Nauertz T, Bernardin L, Unverzagt F. Cognitive dysfunction in multiple sclerosis. II. Impact on employment and social functioning. Neurology. 1991; 41: 692–696. 1823781 10.1212/wnl.41.5.692

[4] Sepulcre J, Vanotti S, Hernández R, Sandoval G, Cáceres F, Garcea O, et al Cognitive impairment in patients with multiple sclerosis using the Brief Repeatable Battery-Neuropsychology test. Mult Scler. 2006; 12: 187–195. doi: 10.1191/1352458506ms1258oa 16629422 10.1191/1352458506ms1258oa

[5] Toledo J, Sepulcre J, Salinas-Alaman A, García-Layana A, Murie-Fernandez M, Bejarano B, et al Retinal nerve fiber layer atrophy is associated with physical and cognitive disability in multiple sclerosis. Mult Scler. 2008; 14: 906–912. doi: 10.1177/1352458508090221 18573835 10.1177/1352458508090221

[6] Batista S, Zivadinov R, Hoogs M, Bergsland N, Heininen-Brown M, Dwyer MG, et al Basal ganglia, thalamus and neocortical atrophy predicting slowed cognitive processing in multiple sclerosis. J Neurol. 2012; 259: 139–146. doi: 10.1007/s00415-011-6147-1 21720932 10.1007/s00415-011-6147-1

[7] Yu HJ, Christodoulou C, Bhise V, Greenblatt D, Patel Y, Serafin D, et al Multiple white matter tract abnormalities underlie cognitive impairment in RRMS. NeuroImage. 2012; 59: 3713–3722. doi: 10.1016/j.neuroimage.2011.10.053 22062194 10.1016/j.neuroimage.2011.10.053

[8] Polman CH, O’Connor PW, Havrdova E, Hutchinson M, Kappos L, Miller DH, et al A randomized, placebo-controlled trial of natalizumab for relapsing multiple sclerosis. N Engl J Med. 2006; 354: 899–910. doi: 10.1056/NEJMoa044397 16510744 10.1056/NEJMoa044397

[9] Radue E-W, Stuart WH, Calabresi PA, Confavreux C, Galetta SL, Rudick RA, et al Natalizumab plus interferon beta-1a reduces lesion formation in relapsing multiple sclerosis. J Neurol Sci. 2010; 292: 28–35. doi: 10.1016/j.jns.2010.02.012 20236661 10.1016/j.jns.2010.02.012

[10] Gunnarsson M, Malmeström C, Axelsson M, Sundström P, Dahle C, Vrethem M, et al Axonal damage in relapsing multiple sclerosis is markedly reduced by natalizumab. Ann Neurol. 2011; 69: 83–89. doi: 10.1002/ana.22247 21280078 10.1002/ana.22247

[11] Miller DH, Soon D, Fernando KT, MacManus DG, Barker GJ, Yousry TA, et al MRI outcomes in a placebo-controlled trial of natalizumab in relapsing MS. Neurology 2007; 68: 1390–1401. doi: 10.1212/01.wnl.0000260064.77700.fd 17452584 10.1212/01.wnl.0000260064.77700.fd

[12] Lublin FD, Cutter G, Giovannoni G, Pace A, Campbell NR, Belachew S. Natalizumab reduces relapse clinical severity and improves relapse recovery in MS. Mult Scler Relat Disord. 2014; 3: 705–711. doi: 10.1016/j.msard.2014.08.005 25891549 10.1016/j.msard.2014.08.005

[13] Kunkel A, Fischer M, Faiss J, Dähne D, Köhler W, Faiss JH. Impact of natalizumab treatment on fatigue, mood, and aspects of cognition in relapsing-remitting multiple sclerosis. Front Neurol. 2015; 6: 97 doi: 10.3389/fneur.2015.00097 26029156 10.3389/fneur.2015.00097 PMC4426783

[14] Matta AP, Nascimento OJ, Ferreira AC, Magalhães TN, Benevides TP, Kirmse A, et al No evidence of disease activity in multiple sclerosis patients. Expert Rev Neurother. 2016; 16: 1279–1284. doi: 10.1080/14737175.2016.1202763 27352830 10.1080/14737175.2016.1202763

[15] Ziemssen T, Derfuss T, de Stefano N, Giovannoni G, Palavra F, Tomic D, et al Optimizing treatment success in multiple sclerosis. J Neurol. 2016; 263: 1053–1065. doi: 10.1007/s00415-015-7986-y 26705122 10.1007/s00415-015-7986-y PMC4893374

[16] Coyle PK, Foley JF, Fox EJ, Jeffery DR, Munschauer FE, Tornatore C. Best practice recommendations for the selection and management of patients with multiple sclerosis receiving natalizumab therapy. Mult Scler. 2009; 15: S26–S36.17.

[17] Plavina T, Subramanyam M, Bloomgren G, Richman S, Pace A, Lee S, et al Anti-JC virus antibody levels in serum or plasma further define risk of natalizumab-associated progressive multifocal leukoencephalopathy. Ann Neurol. 2014; 76: 802–812. doi: 10.1002/ana.24286 25273271 10.1002/ana.24286 PMC4282070

[18] Bloomgren G, Richman S, Hotermans C, Subramanyam M, Goelz S, Natarajan A, et al Risk of natalizumab-associated progressive multifocal leukoencephalopathy. N Engl J Med. 2012; 366: 1870–1880. doi: 10.1056/NEJMoa1107829 22591293 10.1056/NEJMoa1107829

[19] Jeffery DR. Recent advances in treating multiple sclerosis: efficacy, risks and place in therapy. Ther Adv Chronic Dis. 2013; 4: 45–51. doi: 10.1177/2040622312466279 23342246 10.1177/2040622312466279 PMC3539264

[20] Polman CH, Reingold SC, Edan G, Filippi M, Hartung HP, Kappos L, et al Diagnostic criteria for multiple sclerosis: 2005 revisions to the “McDonald Criteria.” Ann Neurol. 2005; 58: 840–846. doi: 10.1002/ana.20703 16283615 10.1002/ana.20703

[21] Kurtzke JF. Rating neurologic impairment in multiple sclerosis: an expanded disability status scale (EDSS). Neurology. 1983; 33: 1444–1452. 6685237 10.1212/wnl.33.11.1444

[22] Layout 1—TOUCH-overview.pdf. https://www.touchprogram.com/TTP/images/TOUCH-overview.pdf. Accessed March 8, 2016.

[23] Smith A, Western Psychological Services (Firm). Symbol Digit Modalities Test. Los Angeles: Western Psychological Services, 1973.

[24] Erlanger DM, Kaushik T, Caruso LS, Benedict RH, Foley FW, Wilken J, et al Reliability of a cognitive endpoint for use in a multiple sclerosis pharmaceutical trial. J Neurol Sci. 2014; 340: 123–129. doi: 10.1016/j.jns.2014.03.009 24656433 10.1016/j.jns.2014.03.009

[25] Smith SM, Zhang Y, Jenkinson M, Chen J, Matthews PM, Federico A, et al Accurate, Robust, and Automated Longitudinal and Cross-Sectional Brain Change Analysis. NeuroImage. 2002; 17: 479–489. 12482100 10.1006/nimg.2002.1040

[26] Smith SM, Jenkinson M, Woolrich MW, Beckmann CF, Behrens TE, Johansen-Berg H, et al Advances in functional and structural MR image analysis and implementation as FSL. NeuroImage. 2004; 23: S208–S219. doi: 10.1016/j.neuroimage.2004.07.051 15501092 10.1016/j.neuroimage.2004.07.051

[27] Patenaude B, Smith SM, Kennedy DN, Jenkinson M. A Bayesian model of shape and appearance for subcortical brain segmentation. NeuroImage. 2011; 56: 907–922. doi: 10.1016/j.neuroimage.2011.02.046 21352927 10.1016/j.neuroimage.2011.02.046 PMC3417233

[28] Battaglini M, Jenkinson M, and De Stefano N. Evaluating and reducing the impact of white matter lesions on brain volume measurements. Hum Brain Mapp. 2012; 33: 2062–2071. doi: 10.1002/hbm.21344 21882300 10.1002/hbm.21344 PMC6870255

[29] Dupuy SL, Tauhid S, Kim G, Chu R, Tummala S, Hurwitz S, et al MRI detection of hypointense brain lesions in patients with multiple sclerosis: T1 spin-echo vs. gradient-echo. Eur J Radiol. 2015; 84:1564–1568. doi: 10.1016/j.ejrad.2015.05.004 26044294 10.1016/j.ejrad.2015.05.004

[30] Fan Q, Teo Y-Y, and Saw S-M. Application of advanced statistics in ophthalmology. Invest Ophthalmol Vis Sci. 2011; 52: 6059–6065. doi: 10.1167/iovs.10-7108 21807933 10.1167/iovs.10-7108

[31] Baroncini D, Ghezzi A, Annovazzi PO, Colombo B, Martinelli V, Minonzio G, et al Natalizumab versus fingolimod in patients with relapsing-remitting multiple sclerosis non-responding to first-line injectable therapies. Mult Scler. 2016; 22: 1315–1326. doi: 10.1177/1352458516650736 27230789 10.1177/1352458516650736

[32] Zivadinov R, Hojnacki D, Bergsland N, Kennedy C, Hagemeier J, Melia R, et al Effect of natalizumab on brain atrophy and disability progression in multiple sclerosis patients over 5 years. Eur J Neurol. 2016; 23: 1101–1109. doi: 10.1111/ene.12992 26998905 10.1111/ene.12992

[33] Ciampi E, Pareto D, Sastre-Garriga J, Vidal-Jordana A, Tur C, Río J, et al Grey matter atrophy is associated with disability increase in natalizumab-treated patients. Mult Scler. 2016 (Forthcoming). 10.1177/1352458516656808 27354019

[34] Alroughani R, Deleu D, El Salem K, Al-Hashel J, Alexander KJ, Abdelrazek MA, et al A regional consensus recommendation on brain atrophy as an outcome measure in multiple sclerosis. BMC Neurol. 2016; 16: 240 doi: 10.1186/s12883-016-0762-5 27881095 10.1186/s12883-016-0762-5 PMC5121973

[35] Shiee N, Bazin PL, Zackowski KM, Farrell SK, Harrison DM, Newesome SD, Rachford JN, et al Revisiting brain atrophy and its relationship to disability in multiple sclerosis. PLoS One. 2012; 7: e37049 doi: 10.1371/journal.pone.0037049 22615886 10.1371/journal.pone.0037049 PMC3352847

[36] Popescu V, Agosta F, Hulst HE, Sluimer IC, Knol DL, Sormani MP, et al Brain atrophy and lesion load predict long term disability in multiple sclerosis. J Neurol Neurosurg Psychiatry 2013; 84: 1082–1091. doi: 10.1136/jnnp-2012-304094 23524331 10.1136/jnnp-2012-304094

[37] Štecková T, Hluštík P, Sládková V, Odstrčil F, Mareš J, Kaňovský P. Thalamic atrophy and cognitive impairment in clinically isolated syndrome and multiple sclerosis. J Neurol Sci. 2014; 342: 62–68. doi: 10.1016/j.jns.2014.04.026 24819917 10.1016/j.jns.2014.04.026

[38] Vollmer T, Huynh L, Kelley C, Galebach P, Signorovitch J, DiBernardo A, et al Relationship between brain volume loss and cognitive outcomes among patients with multiple sclerosis: a systematic literature review. Neurol Sci. 2016; 37: 165–179. doi: 10.1007/s10072-015-2400-1 26537494 10.1007/s10072-015-2400-1

