# Correction: Natalizumab stabilizes physical, cognitive, MRI, and OCT markers of disease activity: A prospective, non-randomized pilot study

**DOI:** 10.1371/journal.pone.0178338

**Published:** 2017-05-18

**Authors:** Garrick D. Talmage, Oscar J. M. Coppes, Adil Javed, Jacqueline Bernard

There are errors in the author affiliations. The affiliations should appear as shown here: Garrick D. Talmage^1^, Oscar J. M. Coppes^2^, Adil Javed^3^, Jacqueline Bernard^4^

**1** Department of Otolaryngology, University of Colorado, Aurora, Colorado, United States of America, **2** Department of Anesthesia, Brigham and Women’s Hospital, Harvard Medical School, Boston, Massachusetts, United States of America, **3** Department of Neurology, The University of Chicago, Chicago, Illinois, United States of America, **4** Department of Neurology, Oregon Health Science University, Portland, Oregon, United States of America.
