# Supplementary material for: Natalizumab stabilizes physical, cognitive, MRI, and OCT markers of disease activity: A prospective, non-randomized pilot study
Source: PLoS One. 2017 Apr 20;12(4):e0173299. doi: 10.1371/journal.pone.0173299 (PMC5398512; doi:10.1371/journal.pone.0173299)
Supplement: S2 File — (DOCX) [file pone.0173299.s002.docx]

Investigator Initiated Trial (IIT)

TYSABRI^®^ (natalizumab)

PROTOCOL TITLE: Tysabri effects on cognition and neurodegeneration

in Multiple Sclerosis

PROTOCOL NUMBER: US-TYS-09-10019

DATE: Final, Feb. 10, 2010

Amendment 1, Mar. 31, 2010

Amendment 2, August 3, 2010

Amendment 3, November 1, 2010

Amendment 4, August 29, 2011

Amendment 5, April 16, 2013

Investigator: Jacqueline T. Bernard, M.D.

Affiliation: University of Chicago, Department of Neurology

TABLE OF CONTENTS

[1.1 Investigator Personnel 4](#_Toc207444787)

[1.2 Biogen Idec, INC. Personnel 5](#_Toc207444788)

[2 ACRONYMS AND ABBREVIATIONS 6](#_Toc207444789)

[3 STUDY FLOW CHART 7](#_Toc207444790)

[4 SUMMARY 8](#_Toc207444791)

[5 Background and Rationale 9](#_Toc207444792)

[6 Methods 11](#_Toc207444793)

[6.1 Study Objectives/Endpoints 11](#_Toc207444794)

[6.2 Study Design 12](#_Toc207444795)

[6.3 Discontinuation of the Study 13](#_Toc207444796)

[7 STUDY POPULATION 14](#_Toc207444797)

[7.1 Number of Subjects 14](#_Toc207444798)

[7.2 Inclusion Criteria 14](#_Toc207444799)

[7.3 Exclusion Criteria 14](#_Toc207444800)

[8 STUDY MEDICATION, DESCRIPTION, AND ALLOCATION 16](#_Toc207444801)

[8.1 Tysabri Stability & Storage 16](#_Toc207444802)

[8.2 Drug Accountability 17](#_Toc207444806)

[8.3 Treatment Schedule for Tysabri 17](#_Toc207444807)

[8.4 Discontinuation of Subjects from Treatment or from the Study…………………………………………………………………………………17](#_Toc207444809)

[8.5 Subject Withdrawal from the Study 18](#_Toc207444810)

[8.6 Treatment Compliance 18](#_Toc207444811)

[8.7 Concomitant Therapy 18](#_Toc207444812)

[8.8 Recording Concomitant Medication 19](#_Toc207444813)

[9 Study Schema 20](#_Toc207444814)

[9.1 Tests and Evaluations 20](#_Toc207444815)

[9.2 Study Site Personnel and Responsibilities 24](#_Toc207444816)

[9.3 Visits and Assessments 26](#_Toc207444817)

[10 Adverse Events/Serious Adverse Events – Definition & reporting 28](#_Toc207444818)

[10.1 Definitions 28](#_Toc207444819)

[10.2 Investigator Responsibilities 31](#_Toc207444820)

[10.3 Additional Procedures 31](#_Toc207444821)

[11 Subject Information and Consent 31](#_Toc207444822)

[11.1 Subject Data Protection 31](#_Toc207444823)

[12 Ethical Requirements 32](#_Toc207444824)

[12.1 Declaration of Helsinki 32](#_Toc207444825)

[12.2 Ethics Committee 32](#_Toc207444826)

[13 Administrative Procedures 32](#_Toc207444827)

[13.1 Patient Enrollment 32](#_Toc207444828)

[13.2 Quality Assurance 32](#_Toc207444829)

[13.3 Study Funding 32](#_Toc207444830)

[14 FURTHER REQUIREMENTS AND GENERAL INFORMATION 33](#_Toc207444831)

[14.1 Internal Service Organizations 33](#_Toc207444832)

[14.2 Study Committees 33](#_Toc207444833)

[14.3 Changes to Final Study Protocol 33](#_Toc207444834)

[14.4 Record Retention 33](#_Toc207444835)

[14.5 Reporting and Communication of Results 34](#_Toc207444836)

[14.6 Protocol Completion 34](#_Toc207444837)

[15 REFERENCES 35](#_Toc207444838)

[16 Appendix I: Signed Agreement of the Study Protocol 37](#_Toc207444839)

[17 Appendix II: World Medical Association Declaration of Helsinki 38](#_Toc207444840)

[18 Appendix III: MEDWATCH Serious Adverse Event Reporting – Form 3500 42](#_Toc207444841)

CONTACT LIST

## Investigator Personnel

Principal Investigator: Jacqueline T. Bernard, M.D.

Office: 773-702-4820

Fax: 773-702-4066

E-Mail: jbernard@neurology.bsd.uchicago.edu

Co-Investigator: Adil Javed, M.D., PhD.

Office: 773-834-0558

Fax: 773-702-9076

E-Mail: ajaved@neurology.bsd.uchicago.edu

Co-Investigator: Anthony Reder, M.D.

Office: 773-702-6204

Fax: 773-702-9076

E-Mail: areder@neurology.bsd.uchicago.edu

Study Coordinator: Christina Sprayberry

Office: 773-834-4654

Fax: 773-834.7801

E-Mail: [cspraybe@neurology.bsd.uchicago.edu](mailto:cspraybe@neurology.bsd.uchicago.edu)

Primary Treating Nurse: Nancy Arndt

Office: 773-795-0107

Fax: 773-8347250

E-Mail: [Nancy.Green3@uchospitals.edu](mailto:Nancy.Green3@uchospitals.edu)

Contract Specialist: Sandy Lieneck

Office: 773-834-1811

Fax: 773-702-9076

E-Mail: slieneck@neurology.bsd.uchicago.edu

## Biogen Idec, INC. Personnel

Biogen Idec

14 Cambridge Center

Cambridge, MA 02142

Medical Science Liaison: Linda A. Piccinini, Ph.D.

Office: 617-230-6015

Fax: 708-358-0948

E-Mail: linda.piccinini@biogenidec.com

# ACRONYMS AND ABBREVIATIONS

**List of abbreviations**

3T 3-Tesla

AAN American Academy of Neurology

AE adverse event

BPF brain parenchymal fraction

BPVC brain percentage volume change

CNS central nervous system

DIR double inverse recovery

DMT disease modifying therapy

DNA Deoxyribonucleic acid

DTI diffusion tensor imaging

EC Ethics Committee

EDSS expanded disability status scale

FDA US Food and Drug Administration

GCP good clinical practice

Gd-DTPA gadolinium diethylenetriamine penta-acetic acid

GM gray matter

IB Investigator’s Brochure

ICF informed consent form

ICH International Conference of Harmonisation

IFN interferon

IV intravenous

IVMP intravenous methylprednisone

IRB Institutional Review Board

MRI magnetic resonance imaging

MS multiple sclerosis

MSFC multiple sclerosis functional composite measure

MTR magnetization transfer ratio

OCT optical coherence tomography

PASAT paced auditory serial addition test

PI principal investigator

PML progressive multifocal leukoencephalopathy

PSIR phase sensitive inversion recovery

RNFL retinal nerve fiber layer

RRMS relapsing-remitting MS

SAE serious adverse event

SDD study drug discontinuation

SDMT symbol digit modalities test

WM white matter

# STUDY FLOW CHART

The Screening phase will be done within 60 days prior to First Dose of Tysabri visit. The Baseline visit assessments may be done up to 7 days prior to the first infusion of Tysabri. All other visit assessment may be done within a 7 day period based on the previous infusion date.

|  | Screening Phase^[[1]](#footnote-1)^ | Wk 0  (Baseline)^[[2]](#footnote-2)^ | Wk 4 | Wk 8 | Wk 12 | Wk 16 | Wk 20 | Wk 24 | Wk 28 | Wk 32 | Wk 36 | Wk 40 | Wk 44 | Wk 48 | Wk 52 | Wk 56 | Wk 60 | Wk 64 | Wk 68 | Wk 72 | Wk 76 | Wk 80 | Wk 84 | Wk 88 | Wk 92 | Wk 96 |
| --- | --- | --- | --- | --- | --- | --- | --- | --- | --- | --- | --- | --- | --- | --- | --- | --- | --- | --- | --- | --- | --- | --- | --- | --- | --- | --- |
| Informed Consent | X |  |  |  |  |  |  |  |  |  |  |  |  |  |  |  |  |  |  |  |  |  |  |  |  |  |
| Inclusion/Exlcusion | X | X |  |  |  |  |  |  |  |  |  |  |  |  |  |  |  |  |  |  |  |  |  |  |  |  |
| Medical History/ Disease History | X |  |  |  |  |  |  |  |  |  |  |  |  |  |  |  |  |  |  |  |  |  |  |  |  |  |
| Physical Examination | X | X |  |  |  |  |  | X |  |  |  |  |  | X |  |  |  |  |  | X |  |  |  |  |  | X |
| EDSS | X | X |  |  |  |  |  | X |  |  |  |  |  | X |  |  |  |  |  | X |  |  |  |  |  | X |
| SDMT |  | X |  |  |  |  |  |  |  |  |  |  |  | X |  |  |  |  |  |  |  |  |  |  |  | X |
| Vital Signs^[[3]](#footnote-3)^ | X | X | X | X | X | X | X | X | X | X | X | X | X | X | X | X | X | X | X | X | X | X | X | X | X | X |
| Urinalysis^[[4]](#footnote-4)^ | X |  |  |  |  |  |  | X |  |  |  |  |  | X |  |  |  |  |  | X |  |  |  |  |  | X |
| Blood draw^[[5]](#footnote-5)^ | X |  |  |  |  |  |  | X |  |  |  |  |  | X |  |  |  |  |  | X |  |  |  |  |  | X |
| Tysabri infusion^[[6]](#footnote-6)^ |  | X | X | X | X | X | X | X | X | X | X | X | X | X | X | X | X | X | X | X | X | X | X | X | X | X |
| Brain MRI Scan ± Gadolinium | X |  |  |  |  |  |  |  |  |  |  |  |  | X |  |  |  |  |  |  |  |  |  |  |  | X |
| RFNL by OCT |  | X |  |  |  |  |  | X |  |  |  |  |  | X |  |  |  |  |  | X |  |  |  |  |  | X |
| Relapse evaluation | X | X |  |  |  |  |  | X |  |  |  |  |  | X |  |  |  |  |  | X |  |  |  |  |  | X |
| Monitoring /Recording of Adverse Events and Concomitant Medication | X | X | X | X | X | X | X | X | X | X | X | X | X | X | X | X | X | X | X | X | X | X | X | X | X | X |

# SUMMARY

**Study Title:** Tysabri effects on cognition and neurodegeneration in Multiple Sclerosis

**Objectives:** The long-term objective is to further establish the role of Tysabri in preventing neurodegeneration in MS and to establish powerful and efficient new surrogate markers for neurodegeneration in MS. It is intended to correlate a single clinical endpoint, cognition, with two outcome measures, using MRI parameters and OCT.

Specific aims:

1. To determine the effects of Tysabri on cognition
2. To determine the effects of Tysabri on MRI surrogate markers of cognitive dysfunction: brain atrophy, cortical atrophy, deep GM atrophy and DTI
3. To determine the effects of Tysabri on RNFL thickness using OCT

**Design:** This is a 96 week, open-label, single center, parallel-group study in 20 subjects with RRMS starting Tysabri treatment

**Patient Population:** The study will be conducted at the University of Chicago. It is aimed to include 20 subjects, male and female between the ages of 18 and 60, who fulfill the inclusion and lack the exclusion criteria.

**Treatment Groups:** Group 1, numbering 10 subjects, includes subjects with MS disease duration less than 2 years and who are naïve to a DMT or have failed 1 DMT. Group 2, numbering 10 subjects, includes subjects with MS disease duration greater than 2 years and who have failed 1 or more DMT. Disease duration is the amount of time from diagnosis.

# Background and Rationale

Asymptomatic, irreversible neurodegeneration can occur early in MS. This can be associated with cognitive dysfunction, which is often underestimated by standard clinical MS screening methods. Patients with MS develop irreversible CNS tissue injury, the end result of which is brain and spinal cord atrophy. Atrophy previously was thought to be a later finding in MS because, despite frequent relapses, patients could remain functionally normal for years after the diagnosis of MS. There currently is a growing literature suggesting that brain atrophy occurs early in MS. Attention has now turned to factors contributing to GM or whole brain as well as WM atrophy. It has been suggested that atrophy measures should be included in clinical trials, and even in clinical practice.

At the time of a first MS attack, patients already have evidence of cognitive dysfunction and atrophy. About 40-65% of MS patients eventually develop cognitive impairment. Often, this can present even before significant physical disability. Patients with MS can be risk stratified to determine the likelihood of later attacks. Patients who have high MRI disease burden and cognitive deficits at presentation are at high risk for future disability and need early treatment.

Studies demonstrate that early diagnosis and intervention can delay progression and disability in MS. We hypothesize that early treatment with Tysabri for MS will delay or reverse parameters of brain atrophy.

There is evidence that IFNs have beneficial effects on cognition and neurodegeneration, but little published data with regard to Tysabri in this area. In the pivotal trial of Interferon-beta-1b, patients receiving treatment had less cognitive dysfunction as measured by PASAT and visual memory scores. Evidence that IFNs slow neurodegeneration was seen in the European secondary progressive trial of interferon-beta-1b. Patients in that study receiving treatment had decreased T1 black hole burden as compared to the placebo group. This suggests that beta-IFNs have a neuroprotective effect, as manifested by less cognitive and physical disability.

There is still a paucity of literature demonstrating that early use of Tysabri can delay cognitive dysfunction and neurodegeneration in MS. To address this, our study proposes to examine the effects of Tysabri on cognitive tests, MRI surrogate markers of cognitive dysfunction, and RNFL measurements as assessed by OCT.

OCT is a non-invasive, objective method, which can provide diagnostic information and quantitative data on biologic tissues with resolution up to 10 microns. The principle of OCT is analogous to ultrasound however the system uses light instead of acoustic waves. Standard OCT scans provide objective and reproducible measurements of the retinal nerve fiber layer (RNFL) thickness. OCT has been shown to have a robust correlation with axonal degeneration in Multiple Sclerosis and may have an application in clinical trials that examine neuroprotective and other disease-modifying therapies.

RNFL measurement by OCT is increasingly being used as a surrogate marker for neurodegeneration. Several studies have shown that RNFL thickness decreases over time as MS continues to progress. Progressive MS has greater reduction in RNFL thickness than early RRMS. There is a correlation between disease stage and level of neurodegeneration as measured by OCT. It is not known whether drug therapy slows RNFL thinning. There is early evidence that IFNs reverse prior deficits, as measured by OCT on RNFL .This study will evaluate whether Tysabri is effective in reducing RNFL thinning, hence providing evidence for neuroprotection.

It is anticipated that every future treatment trial will use RNFL thickness as measured by OCT as an outcome measure. This is in part because of the ease of acquiring data with OCT as well as cost. Data analysis is straightforward. OCT cannot take the place of brain imaging, but provides useful information in conjunction with MRI. This technique can easily be used in the period between brain imaging scans to assess progression.

Although cortical lesions constitute a substantial part of the total lesion load in MS brain, and have been correlated to neuropsychological deficits, the proportion of purely cortical lesions visible on MRI images is not known. They may remain undetected, despite their clinical significance. OCT may detect changes in RNFL and thus axons, which in turn, may correlate with cortical lesions, early in disease, and may better help us find an early window to initiate Tysabri therapy to prevent atrophy and irreversible neurological deficits.

RNFL measurements by OCT are an integral part of this proposal. Several new treatments are expected to become available in the near future. Most have incorporated OCT as secondary outcome measures. Hence, if there were an effect of these agents on OCT measures, then there would be a strong argument for using such agents as first line therapies against MS in order to prevent early neurodegeneration. Hence, it is important to establish the effects of Tysabri on RNFL, which has not been done in a prospective manner so far.

Preliminary Studies/Progress Report

Our group has many years of experience with MS patients and was one of the first to use cognitive assessments in MS clinical trials. Our Brain Research Imaging Center (BRIC) http://bric.bsd.uchicago.edu/ has a newly-installed, state of the art, Phillips 3T magnet, for which its first clinical trial is an industry-supported MS study.

In collaboration with our department Chairman, Christopher Gomez, and his research group, we have launched an IRB approved study of the application of Optical Coherence Tomography (OCT) as a biomarker in neurodegenerative disease. In our initial pilot study of 25 genetically defined subjects with spinocerebellar ataxia and 25 age matched controls, conducted over only 4 days we were able to demonstrate significant differences in RNFL layer thickness using a high-resolution Heidelberg Spectralis OCT machine.

Similarly, we have also just completed a study of 30 Tysabri treated RRMS subjects, looking at RNFL date using Heidelberg Spectralis OCT, the results of which we to submitted to the AAN 2010 national meeting. Some of these subjects also are being studied in parallel with a genomic DNA bank collaboration. We were able to recruit these subjects easily through the MS database at The University of Chicago, and used two existing IRB approved protocols.

In this study, the intention is to establish the role of Tysabri in preventing this early neurodegeneration by showing benefits measured on cognitive testing and radiological measures. We also expect to demonstrate benefit from Tysabri therapy, using OCT as a biomarker for axonal degeneration. We will follow 20 subjects with MS over a two year period.

Other centers that have looked at OCT and RNFL in MS have obtained data using the earlier OCT machines. Heidelberg Spectralis OCT is a newer and more sensitive version, with accuracy to 6 microns and less variability due to the very high level of resolution. This would be one of the first studies in MS using Heidelberg Spectralis OCT.

This study is unique because it uses sensitive clinical and MRI metrics to better define cognitive dysfunction in subjects with RRMS being treated with Tysabri in a prospective manner that has not been studied before. It is anticipated that this study will further underscore the importance of cognitive dysfunction in MS and provide further support for the importance of Tysabri in alleviating cognitive problems in MS.

This study is significant because it proposes to discover and validate new biomarkers (OCT and DTI) that correlate with standard measures of disability and progression. In this way we are applying two new biomarkers to assess their utility in detecting changes that co-vary with cognitive decline and disease progression in the setting of Tysabri therapy. This study will better assess these modalities with regard to their role in the critical issue of neuroprotection in MS.

# Methods

## Study Objectives/Endpoints

Multiple sclerosis is a devastating neurological disease that has benefited significantly by rational drug development and clinical trials using increasingly efficient outcome measures. The long- term objective of the study is to further establish the role of Tysabri in preventing neurodegeneration in MS and to establish powerful and efficient new surrogate markers for neurodegeneration in MS. We intend to correlate a single clinical endpoint, cognition, with two outcome measures, using MRI parameters and OCT.

**Aim 1. To determine the effects of Tysabri on cognition.** We hypothesize that early treatment with Tysabri for MS will delay or reverse cognitive dysfunction. We will test this by following SDMT scores in subjects with RRMS who have received early versus delayed treatment with Tysabri These results will further support the role of Tysabri in protecting cognitive function in MS. Furthermore, these results will help establish a standard upon which to validate the new potential surrogate biomarkers investigated in aims 2 and 3.

**Aim 2. To determine the effects of Tysabri on MRI surrogate markers of cognitive dysfunction: brain atrophy, cortical atrophy, deep gray matter atrophy and DTI**. We hypothesize that early treatment with Tysabri for MS will delay or reverse parameters of brain atrophy. We will accomplish this by comparing brain atrophy, cortical atrophy and DTI in subjects with RRMS who have received early versus late treatment with Tysabri. This will help demonstrate that Tysabri prevents important measures of neurodegeneration.

**Aim 3. To determine the effects of Tysabri on retinal nerve fiber layer (RNFL) thickness using optical coherence tomography (OCT).** In preliminary studies we have utilized a Spectralis high-resolution OCT in the outpatient setting and have been able to efficiently detect significant reductions in RNFL thickness in subjects with neurodegenerative disease compared with age-matched controls. We hypothesize that early treatment with Tysabri for MS will delay or reverse RNFL thinning. We will test this by measuring RNFL thickness-using OCT in subjects with RRMS who have received early versus delayed treatment with Tysabri. These results may establish a new biomarker for neurodegeneration in MS, and for the first time, demonstrate the effect of Tysabri on a new potential biomarker.

We expect to demonstrate that the variables measured in Aims 1-3 correlate with each other, and with primary outcomes of disability, as well as decline in cognition. i.e.: that they have construct validity.

## Study Design

This is a 96 week, open-label, single center, parallel-group study in 20 subjects with RRMS starting Tysabri treatment. The study will be conducted at the University of Chicago. OCT and MRI results collected over two years will be analyzed.

There are two subject groups:

Group 1

10 early diagnosis RRMS subjects that will start Tysabri, are naïve to DMT or have failed 1 DMT and have MS disease duration **less than 2 years** (from diagnosis); will be compared to their own baseline and Group 2.

Group 2

10 later diagnosis RRMS subjects that will start Tysabri, have failed 1 or more DMT and have MS disease duration **more than 2 years** (from diagnosis); will be compared to their own baseline and Group 1.

The study has two Phases: Pre-treatment and Treatment. The Pre-treatment phase consists of two periods, Screening (Day -60 to Day -1, Visit 1) and Baseline (Day -7 to Day -1, Visit 2). Subject eligibility will be assessed during both the Screening and Baseline periods. Eligibility will be assessed based according inclusion/exclusion criteria of the study protocol. Also each subject’s MS activity will be determined by the investigator from the subject’s patient history, past MRI data and past clinical exams.

During the Pre-treatment phase, the subject will be enrolled into the TOUCH® Prescribing Program, as outlined by the FDA guidelines. The subject will also be required to meet any pre-infusion criteria outlined for the TOUCH® program. The study staff will ensure enrollment prior to the first infusion of Tysabri.

Subjects that have been on some types of DMT will require washout-periods (see exclusion criteria for details).

At the First Treatment Visit (Visit 3, Day 1), subjects whose eligibility is confirmed, will receive the first infusion of Tysabri either in the Infusion Center at the University of Chicago or at another outside site of the subject’s choice. Tysabri will be administered by IV at a fixed dose of 300mg every 4 weeks. The infusion will be over one hour. The subject will be monitored for 60 minutes after each infusion. Vitals will be taken prior and post infusion.

The study period for each subject is approximately 96 weeks. Subjects will undergo clinical evaluation (EDSS and physical examination), blood draw, and an OCT evaluation at weeks 24, 48, 72, and 96. At baseline, week 48 and 96 an MRI and SDMT evaluation will be done. Urinalysis will be done at the discretion of the investigator.

The study will terminate when all subjects have completed the 96 week study period. Subjects can continue on Tysabri after the conclusion of the study.

Subjects who suspect they are experiencing new or worsening of symptoms (possible relapse) are to call the *Treating Neurologist* within 48 hours of symptom onset. The *Treating Neurologist* can prescribe treatment of the confirmed relapse with IV methylprednisolone at any time from baseline to Week 96. Treatment with methylprednisolone will not affect the subject’s eligibility to continue in the study.

**Key Efficacy Assessments:** OCT, MRI, EDSS

**Key Safety Assessments:** Physical examination, reporting of adverse events (AEs), infections, vital signs, laboratory analyses, MS relapses.

**Data analysis**: MRI analysis will be done at the University of Chicago using already established commercially available software to assess brain atrophy, cortical atrophy and cortical lesions. The software used will be: FSL, MRIcro, JIM and MEDINRIA. The following measures of atrophy will be used: thalamic and third ventricular volume, BPF, BPVC, T1 black holes, T2 lesion volume, T1 cortical volume (1.5mm slices), DIR, PSIR, and DTI.

The findings of the study will be analyzed using the SPSS V. 17.0 statistical package. Linear correlations between retinal nerve fiber layer thickness and other variables will be evaluated using the Pearson correlation coefficient. Differences between normal (non-MS) controls and the various groups of multiple sclerosis subjects will be assessed using 1-way analysis of variance after screening for homogeneity of variance and normal distributions. Post-hoc comparisons will be made using the Neuman-Kuhls procedure. If the data do not meet the assumptions of parametric tests, differences will be evaluated using non-parametric tests. Probabilities less than 0.05 will be considered statistically significant. It is unknown at this time how many subjects will be needed to show significance, so this is considered a pilot study that could be continued as an extension study.

## Discontinuation of the Study

Biogen Idec, INC. may terminate this study, after consultation with the investigators, at any time.

# STUDY POPULATION

## Number of Subjects

Twenty subjects will be enrolled in the study. If a subject withdraws from the study for any reason, the subject will be replaced by another new consenting subject who meets the criteria, if possible.

## Inclusion Criteria

To be eligible for entry into this study, candidates must meet **all** of the following eligibility criteria at the time of randomization:

1. Male or female

2. 18 through 60 years of age inclusive

3. Diagnosis of relapsing remitting multiple sclerosis as defined by 2005 revised McDonald criteria

4. Prior to treatment phase, have had disease activity with at least:

1 documented relapse during the previous year OR

2 documented relapses during the previous 2 years OR

One or more new MRI lesions (Gd+ and/or T2 hyperintense)

5. An Expanded Disability Status Scale (EDSS) score of 0- 7.0 inclusive

6. Neurologically stable with no evidence of relapse or corticosteroid treatment within 30

days prior to treatment

7. Be naïve to Tysabri.

8. Signed written informed consent prior to participating in the study

## Exclusion Criteria

Candidates will be excluded from study entry if **any** of the following exclusion criteria exist at the time of:

1. A manifestation of MS other than RRMS

2. A history of chronic disease of the immune system other than MS or a known

immunodeficiency syndrome or can be considered by Investigator to be immunocompromised based on subject’s medical history

3. A history or presence of malignancy (except for successfully treated basal or squamous cell carcinoma of skin)

4. Active systemic bacterial, viral or fungal infections, or diagnosis of AIDS, Hepatitis B,

Hepatitis C infection defined as a positive HIV antibody, Hepatitis B surface antigen or

Hepatitis C antibody tests, respectively

5. Have received any live or live attenuated vaccines (including for varicella-zoster virus or

Measles) within 2 months prior to randomization

6. Have received total lymphoid irradiation or bone marrow transplantation

7. Have been treated with:

• corticosteroids or adrenocorticotropic hormones (ACTH) within 1 month prior to

randomization

• IFN-β or glatiramer acetate within 2 weeks prior to randomization

• immunosuppressive medications such as azathioprine or methotrexate within 6

months prior to randomization

• immunoglobulins and/or monoclonal antibodies (including natalizumab) within 6

months prior to randomization

• cladribine, cyclophosphamide or mitoxantrone at any time

8. Any medically unstable condition, as assessed by the primary treating physician

progressive neurological disorder, other than MS, which may affect participation in

the study

9. History of any clinically significant cardiac, endocrinologic, hematologic, hepatic, immunologic, metabolic, urologic, pulmonary, neurologic, dermatologic, psychiatric, renal or other major disease that would preclude participation in a clinical trial (as determined by the Investigator)

10. Unable to undergo MRI scans, including claustrophobia, have a pacemaker or history of hypersensitivity to gadolinium-DTPA

11. Have had a relapse within 30 days prior to Treatment phase AND/OR not stabilized from previous relapse, in the opinion of the Investigator

12. History of severe allergic or anaphylactic reactions or known drug hypersensitivity to natalizumab/Tysabri

13. A clinically significant infectious disease, such as cellulitis, pneumonia, septicemia

14. History of PML

15. Participation in any clinical research study evaluating another investigational drug or

therapy within 6 months prior to randomization

16. History of Tysabri therapy

17. Abnormal screening blood test exceeding any of the limits defined below:

• Alanine transaminase (ALT) or aspartate transaminase (AST) > three times the upper limit of normal (i.e., 3xULN)

• Total white blood cell (WBC) count <2,300/mm^3^

• Platelet count <1000,000/mm^3^

• Creatinine >2xULN

18. Nursing mothers, pregnant women, and women planning to become pregnant while on study.

# STUDY MEDICATION, DESCRIPTION, AND ALLOCATION

The study drug Tysabri will be stored in a secure location, preferably a locked refrigerator. The study drug may be dispensed only by Duchossois Center for Advanced Medicine pharmacy personnel specifically authorized by the investigator.

Study drugs will not to be used beyond the initial expiration date on the drug packaging.

## Tysabri Stability & Storage

TYSABRI® (natalizumab) is a recombinant humanized IgG4k monoclonal antibody produced in murine myeloma cells. Natalizumab contains human framework regions and the

complementarity-determining regions of a murine antibody that binds to a4-integrin. The

molecular weight of natalizumab is 149 kilodaltons. TYSABRI® is supplied as a sterile, colorless, and clear to slightly opalescent concentrate for intravenous (IV) infusion. Each 15 mL dose contains 300 mg natalizumab; 123 mg sodium chloride, USP; 17.0 mg sodium phosphate, monobasic, monohydrate, USP; 7.24 mg sodium phosphate, dibasic, heptahydrate, USP; 3.0 mg polysorbate 80, USP/NF, in water for injection, USP at pH 6.1.

Administration Instructions

Infuse TYSABRI® 300 mg in 100 mL 0.9% Sodium Chloride Injection, USP over approximately one hour. After the infusion is complete, flush with 0.9% Sodium Chloride Injection, USP.

Use of filtration devices during administration has not been evaluated. Other medications should not be injected into infusion set side ports or mixed with TYSABRI®.

TYSABRI® concentrate is supplied as 300 mg natalizumab in a sterile, single-use vial free of preservatives. Each package contains a single-use vial.

Storage

TYSABRI® single-use vials must be refrigerated between 2-8°C (36°-46°F) or stored according to instructions in the Package Inset. Do not use beyond the expiration date stamped on the carton and vial label. DO NOT SHAKE OR FREEZE. Protect from light.

If not used immediately, store the TYSABRI® solution for infusion at 2-8°C (36°-46°F).

TYSABRI® solution for infusion must be administered within 8 hours of preparation.

## Drug Accountability

The study site must maintain accurate records demonstrating dates and amount of study drug received, to whom they are dispensed (subject-by-subject accounting), and accounts of any study drug accidentally or deliberately destroyed.

## Treatment Schedule for Tysabri

Tysabri Tysabri will be infused every four weeks. The scheduling of all visits should be calculated based on the subject’s previous infusion date.

## Discontinuation of Subjects from Treatment or from the Study

The investigator should discontinue study treatment for a given subject or withdraw the

subject from study if it is believed that continuation would be dangerous to the subject’s well-being.

**Study treatment must be *discontinued* under the following circumstances:**

1. The subject becomes pregnant.
2. The subject experiences a medical emergency that warrants permanent discontinuation.
3. At the discretion of the Investigator for medical reasons or for non-compliance.
4. The subject experiences a hypersensitivity reaction to Tysabri.
5. The subject desires to discontinue Tysabri.

Subjects who experience a significant disease progression (1.0 point increase on EDSS that is sustained for 12 weeks or longer) will be notified that they have a worsening of physical disability. These subjects will be informed that they are eligible to discontinue study drug and/or start treatment with interferon-β or glatiramer acetate. All study visits, clinical and MRI evaluations, and safety monitoring will continue as outlined in the protocol.

Subjects who decide to discontinue study drug prematurely but choose to remain in the study, every effort will be made to ensure the subject is followed by the study staff for health and safety reasons. All clinical and MRI evaluations, and safety monitoring will continue as outlined in the protocol. Adverse events and changes in concomitant therapies will continue to be recorded in the subject’s source.

The reason(s) for discontinuing study drug must be recorded in the subject’s source.

## Subject Withdrawal from the Study

Subjects may voluntarily withdraw from the study for any reason at any time. They may be

considered withdrawn if they state an intention to withdraw, fail to return for visits, or become lost to follow-up for any other reason.

If premature withdrawal occurs for any reason, the investigator must make every effort to

determine the primary reason for a subject’s premature withdrawal from the study and record

this information on the source documents.

Subjects may withdraw from the study for any of the following reasons:

1. The subject wished to discontinue participation in the study.
2. The subject is unable or unwilling to follow the protocol.
3. The subject becomes pregnant.

Subjects who withdraw from the study have several options:

1. Subjects may choose to remain on Tysabri, without study evaluations.
2. Subjects may choose to discontinue Tysabri and begin treatment with another approved MS therapy.

The reason(s) for withdrawal from the study must be recorded in the subject’s source.

## Treatment Compliance

Treatment compliance is to be monitored and recorded by staff. Certified infusion center staff will administer the Tysabri infusions. Any and all treatment details will be recorded.

## Concomitant Therapy

A concomitant therapy is any drug or substance taken from the time of the subject’s written consent to until the subject’s final scheduled visit.

There are concomitant therapies not allowed while receiving Tysabri. The following treatments are not allowed:

1. Any alternative drug treatments for MS, such as chronic immunosuppressant therapy or other immunomodulatory treatments. This includes, interferon β, interferon α, Copaxone, cyclophosphamide, methotrexate, azathioprine, 4-aminopyridine or related products.
2. Any investigational product, including investigational therapies for MS or for non-MS.
3. Any systemic steroid therapy, including, oral corticosteroids or periodic treatment with IVMP, except for defined relapses. Steroids that are taken by non-systemic routes, such as topical or inhalation, are allowed.
4. Total lymphoid irradiation, cladribine, T-cell or Tcell receptor vaccination, any therapeutic monoclonal antibody, mitoxantrone, cyclosporine, plasmapheresis, IV immunoglobulin, or cytapheresis.
5. Any live or attenuated vaccine, including for measles.

Subjects who receive any of the above treatments may have to discontinue Tysabri as well as be discontinued from the study.

Symptomatic therapy, such as treatment for spasticity, depression, or fatigue is allowed, but should be given as early as possible during screening, prior to treatment, to maintain consistent treatment for the duration of the study.

Subjects should be told not to start taking any new medications, including over-the-counter drugs, unless they have been permitted by the Investigator.

**Treatment of Relapses on Scheduled or Unscheduled Visits**

A relapse is defined as new or recurring neurologic symptoms not associated with fever or infection that last at least 24 hours and are accompanied by new neurological finding upon examination by the neurologist. New or recurrent neurologic symptoms that evolve gradually over months are considered disease progression and not a relapse, and should not be treated with steroids.

Subjects who suspect they are experiencing new or worsening of symptoms (possible relapse) are to call the *Treating Neurologist* within 48 hours of symptom onset. The *Treating Neurologist* can prescribe treatment of the confirmed relapse with IV methylprednisolone at any time from baseline to Week 96. Treatment with methylprednisolone will not affect the subject’s eligibility to continue in the study.

## Recording Concomitant Medication

The investigator should instruct the subject to notify the study site about any new medications he/she takes after the start of Tysabri. All medications and significant nondrug therapies (including physical therapy and blood transfusions) administered after the subject starts treatment with study drug must be recorded in the source pages.

The use of concomitant therapies defined above must be recorded on the subject’s source records. AEs related to administration of these therapies must be documented as well.

# Study Schema

## Tests and Evaluations

The following test and evaluations will be done throughout the study:

• EDSS

• MRI

• Physical examination

• OCT

• SDMT

• Vital signs, including height and weight

• Urinalysis

• Laboratory evaluations: hematology and chemistry (anti-natalizumab antibody at Week 24, hepatitis B and C and HIV at Screening Visit)

• Pregnancy and assessments of fertility

**EDSS**

(Kurtzke’s) Expanded Disability Status Scale (EDSS) is a scale for assessing neurological impairment in MS. It is a two-part system including (1) a series of scores in each of eight functional systems, and the EDSS steps (ranging from 0 (normal) to 10 (death due to MS). The functional systems are Visual, Brain Stem, Pyramidal, Cerebellar, Sensory, Bowel & Bladder and Cerebral functions. It is recommended that fatigue not to be included into the cerebral score of the EDSS. EDSS will be assessed by the principal investigator or co-investigator at each site, who has successfully completed his/her certification requirements. EDSS assessments are scheduled at baseline, weeks 24, 48, 72 and 96. In case of MS relapse, EDSS assessment is required at every unscheduled visit to confirm relapse.

For all visits, in as much as possible, it is recommended that EDSS assessments be performed

early in the visit, to avoid the subjects from being fatigued during the efficacy assessments

**MRI**

All subjects will undergo magnetic resonance imaging (MRI) scanning of the brain every year, using the same parameters each time.

• Number of *new* [Gd]-enhanced lesions (T1 weighted lesions) compared to the last MRI

• Number of *all* [Gd]-enhanced lesions (T1 weighted lesions)

• Number of new or enlarging T2-weighted lesions compared to the last MRI

• Proportion on subjects without any new MRI disease activity compared to the last MRI

• Combined unique active [MRI] lesions (CUAL) compared to the last MRI

•Thalamic and Third Ventricular Volume

•Brain Parenchymal Fraction (BPF)

•Brain Percentage Volume Change (BPVC)

•T1 black holes

•T2 lesion volume

•T1 Cortical Volume using 1.5 mm slices

•DTI

Each MRI scan performed for the study subject needs to be previewed by a local neuroradiologist. The Primary Treating Physician needs to be contacted in case unexpected findings were detected on MRI scan.

To avoid interferences caused by steroids for the treatment of MS relapses, the following

restrictions apply:

• In case of relapse, if an MRI would have been scheduled within 14 days of the initiation of

steroid treatment, MRI should be performed before steroid treatment is initiated

• No MRI scan should be performed while a subject is on steroid therapy and within the

following 14 days upon termination of steroid therapy as it would be deemed invalid and

not included in the analysis.

As a result of these restrictions, MRI scheduling could be adjusted accordingly.

A designated radiologist or technologist will be maintained as the “site administrator” for all the MRI administrative procedures within the study.

**PHYSICAL EXAMINATION**

A complete physical examination will include the examination of general appearance, skin,

neck (including thyroid), eyes, ears, nose, throat, lungs, heart, abdomen, back, lymph nodes,

extremities, vascular and neurological functions. If indicated based on medical history and/or

symptoms, rectal, external genitalia, breast, and pelvic exams will be performed. Information for all physical examinations must be included in the source documentation. **Significant findings that are present prior to the start of study drug must be included in the Relevant Medical History/Current Medical Conditions screen.** Significant findings made after the start of study drug which meet the definition of an Adverse Event must be recorded on the Adverse Event source page.

**OCT**

Retinal nerve fiber layer thickness (RNFL) will be measured at baseline, weeks 24, 48, 72 and 96.using the Heidelberg Spectralis OCT machine.

**SDMT**

 The *Symbol Digit Modalities Test* (SDMT) detects cognitive impairment. The SDMT is sensitive in detecting not only the presence of brain damage, but also changes in cognitive functioning over time and in response to treatment.

The SDMT involves a simple substitution task. Using a reference key, the examinee has 90 seconds to pair specific numbers with given geometric figures. Responses can be written or oral, and for either response mode, administration time is 5 minutes.

Individuals with cerebral dysfunction perform poorly on the SDMT, in spite of normal or above average intelligence.

**VITAL SIGNS**

Vital signs include blood pressure (systolic and diastolic), pulse measurements, and temperature (degrees Celsius).

**WEIGHT**

Body weight (to the nearest 0.1 kilogram [kg] in indoor clothing, but without shoes) will be measured.

**LABORATORY EVALUATIONS**

Analysis of all specimens collected will be done at the central laboratory of the Medical Center at the University of Chicago.

The results of the analysis will be made available on the hospital’s on-line EPIC system. Extreme values will generate an “alert” to the investigator. Investigators will be asked to comment on these abnormalities on the respective lab result page, including a notation of the clinical significance of each abnormal finding in the subject’s source documents.

Abnormal laboratory parameters, inconsistent with clinical presentation of MS or

suspicious of underlying medical condition, should be repeated for accuracy.

Abnormal laboratory values should not be recorded on the AE/Infection source page; however, any diagnoses (or signs or symptoms if a diagnosis is not possible) associated with the abnormal findings should be recorded on the AE/Infection source page.

Clinically notable laboratory findings are defined by the hospital standards.

Hematology parameters will be collected at each scheduled visit and will include: red blood

cell (RBC) count, total and differential WBC count (basophiles, eosinophils, lymphocytes,

monocytes, neutrophils, WBC segments), platelet count, hemoglobin, hematocrit, MCV,

MCH, MCHC and RBC morphology.

Blood chemistry (serum) samples will include the following parameters: electrolytes (Na, K, Cl, bicarbonate, Ca, Mg, P), random glucose, fasting glucose, albumin, alkaline phosphatase, creatinine, ALT, AST, GGT, amylase, total bilirubin, conjugated bilirubin, total cholesterol, CRP, triglycerides, HDL, LDL. If an increase of amylase above the clinically notable value is observed (≥ 300U/L), a lipase should be tested to determine the origin of elevated amylase (pancreatic versus extra pancreatic).

At the Screening Visit, the blood draw analysis will include hepatitis B (hepatitis B surface antigen [HBsAg] and/or hepatitis B core antibody [HBcAb], hepatitis C (hepatitis C virus antibody [HCV Ab]) and HIV. Subjects with a positive test result for any of these viruses will be excluded. Also should a subject test positive, information about the person will be disclosed to the Illinois Department of Public Health as mandated.

At the Week 24 the blood draw will include the anti-natalizumab antibody analysis to ensure that the subject is not becoming immune to the drug and its effects.

A serum pregnancy test will be performed for any female subject with a positive urinalysis pregnancy test. Subject s that have been confirmed to be pregnant will be permanently discontinued.

**URINALYSIS**

Dipstick measurements for protein, bilirubine, ketones, pH, urobilinogen, glucose and blood

will be performed. WBC and RBC sediments will also be measured. Urinalysis will be done at the discretion of the investigator. Any female subject will have at least 1 urinalysis pregnancy test prior to her first infusion of Tysabri.

**PREGNACY**

Additional pregnancy testing may be performed at the investigator’s discretion during the

study. Subjects becoming pregnant must permanently discontinue the study drug.

## Study Site Personnel and Responsibilities

**Role of Key Site personnel**

In order to facilitate the required study conduct at each investigational site, it is essential that

the Primary Investigator, a neurologist, assigns site personnel to the following roles:

• **Primary Treating Physician**, a physician with appropriate clinical experience in

neurology. The Primary Investigator usually serves as the Primary Treating Physician

• **Primary Treating Nurse**

• **Study Coordinator**

• **MRI technician**

• **Neuroradiologist**

• **Pharmacist**

Where specified, evaluations described in the protocol must be performed only by the personnel indicated. Plans for appropriate backup personnel for Primary Treating Physician

need to be arranged in advance.

**Primary Treating Physician**

The Primary Treating Physician will be responsible for:

• Overall conduct of the study at the study site

• Management of the routine clinical care of the study subjects

• Confirmation of subject’s eligibility for Treatment phase

• Obtaining the EDSS score, based on a detailed neurological examination as per protocol

schedule

• Assessment and treatment of adverse events and MS attacks/relapses should they occur

• Monitoring of adverse events in subjects who have permanently discontinued study drug, etc.

• Ensuring that all treating site personnel are informed of concomitant medications excluded

per protocol (e.g. immunosuppressive therapy other than steroids used for the treatment of

MS attacks/relapses)

• Ensuring that the need for increased vigilance regarding infections during and in the

weeks following administration of corticosteroids is explained to the subject

• To ensure that all subjects have a Patient Information Card (with site contact information

and which identifies them as participants in a clinical study taking Tysabri, a drug with potential immunosuppressive effects) and to instruct them to show this card to any local physician/health care provider they may consult.

• Reminding subjects of the following at each visit:

• To be alert to symptoms of infections, especially during steroid therapy and in the weeks thereafter, and to promptly report these symptoms

• The importance of informing all health care providers they may visit that they are in a

study and are receiving Tysabri, a drug that suppresses their immune response

**Study Coordinator**

The Study Coordinator will be responsible for:

• Assisting the treating neurologist in subject management, including the assessment and

treatment of adverse events and disease relapses and the recording of adverse events and

concomitant medications.

• Scheduling visits and assessments as outlined in the study protocol, maintaining proper

source documentation and transcription of the data to the CRFs.

• Coordination with and between the study selected central labs.

• Assisting with the completion of visit assessments, i.e. SDMT, OCT, etc.

**Primary Treating Nurse**

A Primary Treating Nurse will be responsible for:

• Subject’s enrollment into the TOUCH® program

• Administer and/or supervise Tysabri infusions.

• Clinically monitor the subject for at least 60 minutes post-dose, record vital signs and/or AEs.

• Review vital signs and make a decision regarding subjects’ eligibility for discharge.

• Provide instructions to the subject upon discharge

• Maintain and ensure accuracy of completeness of source records during dose monitoring.

**MRI technician**

An MRI technician will be responsible for:

• Familiarization with the MRI manual procedures and the study specific MRI protocol, and

loading the MRI parameters on the study designated MRI scanner prior to the subject

enrollment.

• Performance of a “dry” or “dummy” run using the MRI parameters outlined by the MRI

protocol.

• Accommodation of subjects for the MRI scan as per request of the treating nurse and

performance of high-quality MRI scans using the stored parameters in the designated MRI

scanner for the duration of the study.

• Providing appropriate MRI data as outlined by the MRI protocol immediately upon

completion of each MRI scan.

**Neuroradiologist**

A neuroradiologist will be responsible for:

• Review of each MRI scan performed for the study subjects at the site.

• Contacting the Primary Treating Physician only in case of unexpected, not MS related

findings detected on the MRI scan.

**Pharmacist**

The Pharmacist will be responsible for:

• Storage and distribution of Tysabri to Treating Nurse.

## Visits and Assessments

On Baseline visit, all tests and assessments must be performed prior to subject receiving the first dose of Tysabri.

Unscheduled neurological worsening visits should occur if any new neurological symptom(s) that suggest neurological worsening or a possible relapse.

**SCREENING VISIT (within 60 days prior to first infusion)**

The following assessments will be done at the Screening Visit:

• Informed consent

• Eligibility criteria

• Medical history

• Physical examination

• EDSS

• Vital signs, including height and weight

• Urinalysis, at the discretion of the investigator

• Blood draw (hematology, hepatitis B and C, HIV, and serum chemistry)

• Pregnancy test if applicable

• MRI assessment

• Recording of concomitant therapies

**BASELINE VISIT/WEEK 0 (within 7 days of first infusion)**

The following assessments will be done at the Baseline Visit:

• Eligibility criteria

• Physical examination

• EDSS

• SDMT

• Vital signs, including weight

• Urinalysis, at the discretion of the investigator

• Pregnancy test if applicable

• RNFL thickness assessment by OCT (may also be done at screening visit)

• Recording of concomitant therapies and AEs if applicable

• Relapse evaluation

• Tysabri infusion, after all other assessments are done

**WEEK 4, 8, 12, 16, 20 (each visit +/- 7 days)**

• Vital signs

• Recording of concomitant therapies and AEs if applicable

• Relapse evaluation

• Tysabri infusion

**WEEK 24 VISIT (visit +/- 7 days)**

The following assessments will be done at the Week 24 Visit:

• Physical examination

• EDSS

• Vital signs, including weight

• Urinalysis, at the discretion of the investigator

• Blood draw (hematology, serum chemistry, AND anti-natalizumab antibody)

• RNFL thickness assessment by OCT

• Recording of concomitant therapies and AEs if applicable

• Relapse evaluation

• Tysabri infusion, after all other assessments are done

**WEEK 28, 32, 36, 40, 44 (each visit +/- 7 days)**

• Vital signs

• Recording of concomitant therapies and AEs if applicable

• Relapse evaluation

• Tysabri infusion

**WEEK 48 VISIT (visit +/- 7 days)**

The following assessments will be done at the Week 48 Visit:

• Physical examination

• EDSS

• SDMT

• Vital signs

• Weight

• Urinalysis, at the discretion of the investigator

• Blood draw (hematology and serum chemistry)

• RNFL thickness assessment by OCT

• MRI assessment

• Recording of concomitant therapies and AEs if applicable

• Relapse evaluation

• Tysabri infusion, after all other assessments are done

**WEEK 52, 56, 60, 64, 68 (each visit +/- 7 days)**

• Vital signs

• Recording of concomitant therapies and AEs if applicable

• Relapse evaluation

• Tysabri infusion

**WEEK 72 VISIT (visit +/- 7 days)**

The following assessments will be done at the Week 72 Visit:

• Physical examination

• EDSS

• Vital signs

• Weight

• Urinalysis, at the discretion of the investigator

• Blood draw (hematology and serum chemistry)

• RNFL thickness assessment by OCT

• Recording of concomitant therapies and AEs if applicable

• Relapse evaluation

• Tysabri infusion, after all other assessments are done

**WEEK 76, 80, 84, 88, 92 (each visit +/- 7 days)**

• Vital signs

• Recording of concomitant therapies and AEs if applicable

• Relapse evaluation

• Tysabri infusion

**WEEK 96 VISIT (visit +/- 7 days)**

The following assessments will be done at the Week 96 Visit:

• Physical examination

• EDSS

• SDMT

• Vital signs

• Weight

• Urinalysis, at the discretion of the investigator

• Blood draw (hematology and serum chemistry)

• RNFL thickness assessment by OCT

• MRI assessment

• Recording of concomitant therapies and AEs if applicable

• Relapse evaluation

• Tysabri infusion, after all other assessments are done

# Adverse Events/Serious Adverse Events – Definition & reporting

At the signing of the informed consent form, each subject will be given the names and telephone numbers of investigational site personnel for reporting adverse events and medical emergencies. Definitions and instructions for monitoring, recording, and reporting adverse events will be reviewed with investigational site personnel prior to enrollment.

## Definitions

### Adverse Event

Any untoward medical occurrence in a patient or clinical investigation subject administered a pharmaceutical product and which does not necessarily have to have a causal relationship with this treatment. An adverse event (AE) is any adverse event that is experienced by a subject who has received a medicinal drug, but that does not necessarily have a causal relationship with the medicinal drug.

### Serious Adverse Events

In accordance with 21 Code of Federal Regulations (CFR) Part 312.32 and the recommendations of the International Conference on Harmonisation (ICH) [Federal Register, October 7, 1997, Vol. 62, No. 194, pp 52239-45], any of the following adverse events are to be classified as a serious adverse event (SAE):

- An event that results in death.
- An event that, in the view of the Investigator, places the subject at immediate risk of death (a life-threatening event). This does not include an event that, had it occurred in a more severe form, might have caused death.
- An outcome that results in a congenital anomaly/birth defect diagnosed in a child of a subject who participated in this study.
- An event that requires or prolongs in-patient hospitalization.
- An event that results in persistent or significant disability/incapacity.
- Other medically important events that, in the opinion of the Investigator, may jeopardize the subject or may require intervention to prevent one of the other outcomes listed in the definition above. (Examples of such medical events include allergic bronchospasm requiring intensive treatment in an emergency room or convulsions occurring at home that do not require an in-patient hospitalization.).

*If a serious adverse event is unresolved when a subject permanently discontinues the study, the subject will be followed until the event resolves or the clinical course is stabilized.*

### Adverse Event Recording/Reporting

All adverse events (including pre-dosing and treatment-emergent) should be recorded in the subject’s record (or, if applicable, in the adverse event section of the CRF) regardless of severity or relationship to study treatment.

### Immediate Reporting of Serious Adverse Events

| Any SAE required to be reported according to 21 Code of Federal Regulations (CFR) Part 312.32 and the recommendations of the International Conference on Harmonisation (ICH) [Federal Register, October 7, 1997, Vol. 62, No. 194, pp 52239-45] that occurs regardless of whether or not the subject has undergone any study-related procedures or received medicinal drug, through the completion of trial, will be reported to the FDA.  ***The Investigator will notify the local IRB/IEC per local requirements.*** ***The investigator may also notify Biogen Idec, INC of any major safety event AFTER the event is submitted to the FDA and the study IRB/IEC.*** |
| --- |

### Safety Classifications

Site Investigator or Designee:

Record event in subject's CRF as per CRF instructions

The following classifications should be considered when evaluating the relationship of adverse events and serious adverse events to the study treatment:

| *Relationship of Event to Study Treatment* | |
| --- | --- |
| Not related | An adverse event will be considered “not related” to the use of the study treatment if there is not a possibility that the event has been caused by the product. Factors pointing toward this assessment include, but are not limited to: the lack of reasonable temporal relationship between administration of the drug and the event, the presence of a biologically implausible relationship between the product and the adverse event (e.g., the event occurred before administration of drug), or the presence of a more likely alternative explanation for the adverse event. |
| Related | An adverse event will be considered “related” to the use of the study treatment if there is a possibility that the event may have been caused by the product. Factors that point toward this assessment include, but are not limited to: a positive re-challenge, a reasonable temporal sequence between administration of the drug and the event, a known response pattern of the suspected drug, improvement following discontinuation or dose reduction, a biologically plausible relationship between the drug and the adverse event, or a lack of an alternative explanation for the adverse event. |
|  |  |
|  |  |

Severity of Adverse Events and Serious Adverse Events

The following classifications should be considered when evaluating the severity of adverse events and serious adverse events:

| ***Severity of Event*** | |
| --- | --- |
| Mild: | Symptoms(s) barely noticeable to subject or does not make subject uncomfortable; does not influence performance or functioning; prescription drug not ordinarily needed for relief of symptoms(s) but may be given because of personality of subject. |
| Moderate: | Symptom(s) of a sufficient severity to make subject uncomfortable; performance of daily activity is influenced; subject is able to continue in study; treatment for symptom(s) may be needed. |
| Severe: | Symptom(s) cause severe discomfort; symptoms cause incapacitation or significant impact on subject’s daily life; severity may cause cessation of treatment with medicinal drug; treatment for symptom(s) may be given and/or subject hospitalized. |

## Investigator Responsibilities

The Investigator will:

- Monitor and record all adverse events
- Determine the seriousness, causality, and severity of each adverse event
- Report all serious adverse events to the FDA according to the code of federal regulations
- Actively and persistently pursue follow-up of serious adverse events
- Notify Biogen Idec, INC. of any major safety event

## Additional Procedures

### Procedures for Handling Pregnancy

TYSABRI® (natalizumab) treatment will be immediately discontinued in the event of pregnancy in a subject enrolled in the study.

# Subject Information and Consent

Prior to any testing under this protocol, including screening tests and evaluations, all subjects will sign an informed consent form that complies with the requirements of both 21 CFR Part 50 and HIPAA before entering the study. Or, a consent form that complies with the requirements of 21 CFR Part 50 and a separate HIPAA compliant authorization form for the use of and disclosure of the subject’s protected health information (PHI) will be obtained from the subject in accordance with local practice and regulations.

The background of the proposed study and the benefits and risks of the procedures and study will be explained to the subject. A copy of the informed consent document signed and dated by the subject will be given to the subject*.* Confirmation of a subject’s informed consent will also be documented in the subject’s medical records prior to any testing under this protocol, including screening tests and evaluations.

## Subject Data Protection

The subject will not be identified by name in any study reports, and these reports will be used for research purposes only. Every effort will be made to keep the subject’s personal medical data confidential.

# Ethical Requirements

The Investigator must comply with all instructions, regulations, and agreements in this protocol and in the applicable ICH and GCP guidelines, and must also conduct the study according to local regulations.

## Declaration of Helsinki

The Investigator must follow the recommendations contained in the Declaration of Helsinki, amended at the 52nd WMA General Assembly, Edinburgh, Scotland, October 2000, with Note of Clarification on Paragraph 29 added by the WMA General Assembly, Washington 2002. See Appendix II in Section 15.2.

## Ethics Committee

The Investigator must obtain written IRB approval of the protocol, ICF, and other required study documents prior to starting the study.

# Administrative Procedures

## Patient Enrollment

The Investigator must not enroll any subjects into the study prior to all prerequisite study document completion and agreement by the Investigator and Biogen Idec, INC.

## Quality Assurance

During and/or after completion of the study, quality assurance officers named by Biogen Idec, INC. or the regulatory authorities may wish to perform on-site audits. The Investigator will be expected to cooperate with any audit and to provide assistance and documentation (including source data) as requested.

## Study Funding

Biogen Idec, INC. will financially support the work of the Investigator as it pertains to the conduct of this study. All financial details are provided in the separate contract between the Investigator and Biogen Idec, INC.

# FURTHER REQUIREMENTS AND GENERAL INFORMATION

## Internal Service Organizations

### On-site Laboratories for Laboratory Evaluations

Any laboratory evaluations will be done on site at the central laboratory for the University of Chicago Medical Center.

### OCT Reading Center

MRI evaluations will be done at the Brain and Research Imaging Center in the Bernard Mitchell Hospital at the University of Chicago. All scans will be read by the MRI neuroradiologist for assessment of any non-MS pathology. Original MRI images will be reviewed and analyzed on site using commercially available software (FSL, MRIcro, JIM and MEDINRIA).

### Data Monitoring Committee

Data will be reviewed once a year for safety monitoring by the PI and two of the co-investigators, all three of personnel being neurologists. A report will be generated, kept on file and submitted to the local IRB at the time of the annual continuing review.

## Study Committees

### Publication Policy

Biogen Idec, INC. reserves the right to a 60-day review of all manuscripts related to the study prior to publication. Investigators should refer to their Clinical Trial Agreement for additional details regarding the disclosure of study results.

## Changes to Final Study Protocol

All protocol amendments must be submitted to the IRB/EC. Protocol modifications that impact subject safety, the scope of the investigation, or the scientific quality of the study must be approved by the IRB/EC and submitted to the appropriate regulatory authorities (if applicable) before implementation of such modifications to the conduct of the study. In the event of a protocol modification, the subject consent form may also require modifications.It is the responsibility of the PI to submit all revisions to the protocol to Biogen Idec, INC.

## Record Retention

Appropriate legal guidelines regarding retention of records must be followed.

## Reporting and Communication of Results

No publication or disclosure of study results will be permitted except as specified in a separate, written agreement between Biogen Idec, INC. and the Investigator.

## Protocol Completion

The IRB/EC must be notified of completion or termination of the protocol. Within 3 months of protocol completion or termination, the investigator must provide a final clinical summary report to the IRB/EC. The principal investigator will maintain an accurate and complete record of all submissions made to the IRB/EC, including a list of all reports and documents submitted. A copy of these reports will be sent to Biogen Idec, INC. Adverse events, which are reported to regulatory authorities, must be submitted promptly to the IRB/EC.

# REFERENCES

Rudick, Richard. Brain Atrophy Occurs Earlier Than Expected in MS. Journal Watch Neurology 2002.

Geurts, J., Bo, L., Pauwels, P., Castelinjns, J., Polman, C., Barkhof, F. Cortical Lesions in Multiple Sclerosis: Combined Postmortem MR Imaging and Histopathology. AJNR 26:572-577, 2005.

Fisher, J., Jacobs, D., Markowitz, C., Galetta, S., Volpe, N., Nano-Schiavi, M., Baier, M., Frohman, E., Winslow, H., Frohman, T., Calabresi, P., Maguire, M., Cutter, G., Balcer, L. Relation of visual function to retinal nerve fiber layer thickness in multiple sclerosis. Ophthalmology 113:324-332, 2006.

Charil, A., Dagher, A., Lerch, J., Zijdenbos, A., Worslev, K., Evans, A. Focal cortical atrophy in multiple sclerosis: relation to lesion load and disability. NeuroImage 34:509-517, 2007.

Gordon-Lipkin, E., Chodkowski, B., Reich, D., Smith, S., Pulicken, M., Balcer, L., Frohman, E., Cutter, G., Calabresi P. Retinal nerve fiber layer is associated with brain atrophy in multiple sclerosis. Neurology 69:1603-1609, 2007.

Parmenter, B.,Weinstock-Guttman, B., Garg, N., Munschauer, F., Benedict, R. Screening for cognitive impairment in multiple sclerosis using the Symbol Digit Modalities Test. Mult Scler 13: 52-57, 2007.

Gordon-Lipkin, E., Chodkowski, B., Reich, D., Smith, S., Pulicken, M., Balcer, L., Frohman, E., Cutter, G., Calabresi, P. Retinal nerve fiber layer is associated with brain atrophy in multiple sclerosis. NEUROLOGY 69:1603-1609, 2007.

Frohman, E., Fujimoto, J., Frohman, T., Calabresi, P., Cutter G., Balcer L. Optical coherence tomography: a window into the mechanisms of multiple sclerosis. Nat Clin Pract Neurol 4:664-675, 2008.

Siger, M., Dziegielewshi, K., Jasek, L., Bieniek, M., Nicpan, A., Nawrocki, J., Selmaj, K. Optical coherence tomography in multiple sclerosis: thickness of the retinal nerve fiber layer as a potential measure of axonal loss and brain atrophy. J Neurol 255:1555-1560, 2008.

Toledo, J., Sepulcre, J., Salinas-Alaman, A., Garcia-Layana, A., Murie-Fernandez, M., Bejarano, B., Villoslada, P. Retinal nerve fiber layer atrophy is associated with physical and cognitive disability in multiple sclerosis. Mult Scler 14:906-912, 2008.

Cettomai, D., Pulicken, M., Gordon-Lipkin, E., Salter, A., Frohman, T., Conger, A., Zhang, X., Cutter, G., Balcer, L., Frohman, E., Calabresi, P. Reproducibility of optical coherence tomography in multiple sclerosis. Arch Neurol 65:1218-1222, 2008.

Fong, J., Rae-Grant, A., Huang, D. Neurodegeneration and neuroprotective agents in multiple sclerosis. Recent Pat CNS Drug Discov 3:153-165, 2008.

Frohman, E., Dwyer, M., Frohman, T., Cox, J., Salter, A., Greenberg, B., Hussein, S., Conger A., Calabresi P., Balcer, L., Zivadinov, R. Relationship of optic nerve and brain conventional and nonconventional MRI measures and retinal nerve fiber layer thickness, as assessed by OCT and GDx: A pilot study. J Neurol Sci 282:96-105, 2009.

Barkhof, F., Calabresi, P., Miller, D., Reingold, S. Imaging outcomes for neuroprotection and repair in multiple sclerosis trials. Nature 5:256-266, 2009.

Rudick, R., Trapp, B. Gray-Matter Injury in Multiple Sclerosis. N Engl J Med 361:1505-1506, 2009.

Wolinsky, J. MRI lesions loads and disability relationships in MS. More similar than different? Neurology 14. 2009.

Hasan, K., Halphen, C., Kamali, A., Nelson, F., Wolinsky, J., Narayana, P. Caudate nuclei volume, diffusion tensor metrics, and T(2) relaxation in healthy adults and relapsing-remitting multiple sclerosis patients: implications for understanding gray matter degeneration. J Magn Reson Imaging 29(1):70-77, 2009.

Kurtzke JF, MD (1983) Rating neurologic impairment in multiple sclerosis: An expanded

disability status scale (EDSS). Neurology; 33: 1444-1452.

# Appendix I: Signed Agreement of the Study Protocol

I have read the foregoing protocol, entitled ‘Tysabri effects on cognition and neurodegeneration in Multiple Sclerosis” and agree to conduct the study as detailed herein and to inform all who assist me in the conduct of this study of their responsibilities and obligations.

Principal Investigator's Signature Date

Jacqueline T. Bernard, M.D.

Principal Investigator's Name (Print)

University of Chicago

Investigational Site (Print)

# Appendix II: World Medical Association Declaration of Helsinki

**Ethical Principles for Medical Research Involving Human Subjects**

Adopted by the 18th WMA General Assembly

Helsinki, Finland, June 1964

and amended by the

29th WMA General Assembly, Tokyo, Japan, October 1975

35th WMA General Assembly, Venice, Italy, October 1983

41st WMA General Assembly, Hong Kong, September 1989

48th WMA General Assembly, Somerset West, Republic of South Africa, October 1996

and the

52nd WMA General Assembly, Edinburgh, Scotland, October 2000

A. INTRODUCTION

1. The World Medical Association has developed the Declaration of Helsinki as a statement of ethical principles to provide guidance to physicians and other participants in medical research involving human subjects. Medical research involving human subjects includes research on identifiable human material or identifiable data.
2. It is the duty of the physician to promote and safeguard the health of the people. The physician's knowledge and conscience are dedicated to the fulfillment of this duty.
3. The Declaration of Geneva of the World Medical Association binds the physician with the words, “The health of my patient will be my first consideration,” and the International Code of Medical Ethics declares that, “A physician shall act only in the patient's interest when providing medical care which might have the effect of weakening the physical and mental condition of the patient.”
4. Medical progress is based on research, which ultimately must rest in part on experimentation involving human subjects.
5. In medical research on human subjects, considerations related to the well-being of the human subject should take precedence over the interests of science and society.
6. The primary purpose of medical research involving human subjects is to improve prophylactic, diagnostic and therapeutic procedures and the understanding of the aetiology and pathogenesis of disease. Even the best proven prophylactic, diagnostic, and therapeutic methods must continuously be challenged through research for their effectiveness, efficiency, accessibility and quality.
7. In current medical practice and in medical research, most prophylactic, diagnostic and therapeutic procedures involve risks and burdens.
8. Medical research is subject to ethical standards that promote respect for all human beings and protect their health and rights. Some research populations are vulnerable and need special protection. The particular needs of the economically and medically disadvantaged must be recognized. Special attention is also required for those who cannot give or refuse consent for themselves, for those who may be subject to giving consent under duress, for those who will not benefit personally from the research and for those for whom the research is combined with care.
9. Research Investigators should be aware of the ethical, legal and regulatory requirements for research on human subjects in their own countries as well as applicable international requirements. No national ethical, legal or regulatory requirement should be allowed to reduce or eliminate any of the protections for human subjects set forth in this Declaration.

B. BASIC PRINCIPLES FOR ALL MEDICAL RESEARCH

1. It is the duty of the physician in medical research to protect the life, health, privacy, and dignity of the human subject.
2. Medical research involving human subjects must conform to generally accepted scientific principles, be based on a thorough knowledge of the scientific literature, other relevant sources of information, and on adequate laboratory and, where appropriate, animal experimentation.
3. Appropriate caution must be exercised in the conduct of research, which may affect the environment, and the welfare of animals used for research must be respected.
4. The design and performance of each experimental procedure involving human subjects should be clearly formulated in an experimental protocol. This protocol should be submitted for consideration, comment, guidance, and where appropriate, approval to a specially appointed ethical review committee, which must be independent of the investigator, the sponsor or any other kind of undue influence. This independent committee should be in conformity with the laws and regulations of the country in which the research experiment is performed. The committee has the right to monitor ongoing trials. The researcher has the obligation to provide monitoring information to the committee, especially any serious adverse events. The researcher should also submit to the committee, for review, information regarding funding, sponsors, institutional affiliations, other potential conflicts of interest and incentives for subjects.
5. The research protocol should always contain a statement of the ethical considerations involved and should indicate that there is compliance with the principles enunciated in this Declaration.
6. Medical research involving human subjects should be conducted only by scientifically qualified persons and under the supervision of a clinically competent medical person. The responsibility for the human subject must always rest with a medically qualified person and never rest on the subject of the research, even though the subject has given consent.
7. Every medical research project involving human subjects should be preceded by careful assessment of predictable risks and burdens in comparison with foreseeable benefits to the subject or to others. This does not preclude the participation of healthy volunteers in medical research. The design of all studies should be publicly available.
8. Physicians should abstain from engaging in research projects involving human subjects unless they are confident that the risks involved have been adequately assessed and can be satisfactorily managed. Physicians should cease any investigation if the risks are found to outweigh the potential benefits or if there is conclusive proof of positive and beneficial results.
9. Medical research involving human subjects should only be conducted if the importance of the objective outweighs the inherent risks and burdens to the subject. This is especially important when the human subjects are healthy volunteers.
10. Medical research is only justified if there is a reasonable likelihood that the populations in which the research is carried out stand to benefit from the results of the research.
11. The subjects must be volunteers and informed participants in the research project.
12. The right of research subjects to safeguard their integrity must always be respected. Every precaution should be taken to respect the privacy of the subject, the confidentiality of the patient's information and to minimize the impact of the study on the subject's physical and mental integrity and on the personality of the subject.
13. In any research on human beings, each potential subject must be adequately informed of the aims, methods, sources of funding, any possible conflicts of interest, institutional affiliations of the researcher, the anticipated benefits and potential risks of the study and the discomfort it may entail. The subject should be informed of the right to abstain from participation in the study or to withdraw consent to participate at any time without reprisal. After ensuring that the subject has understood the information, the physician should then obtain the subject's freely-given informed consent, preferably in writing. If the consent cannot be obtained in writing, the non-written consent must be formally documented and witnessed.
14. When obtaining informed consent for the research project the physician should be particularly cautious if the subject is in a dependent relationship with the physician or may consent under duress. In that case the informed consent should be obtained by a well-informed physician who is not engaged in the investigation and who is completely independent of this relationship.
15. For a research subject who is legally incompetent, physically or mentally incapable of giving consent or is a legally incompetent minor, the investigator must obtain informed consent from the legally authorized representative in accordance with applicable law. These groups should not be included in research unless the research is necessary to promote the health of the population represented and this research cannot instead be performed on legally competent persons.
16. When a subject deemed legally incompetent, such as a minor child, is able to give assent to decisions about participation in research, the investigator must obtain that assent in addition to the consent of the legally authorized representative.
17. Research on individuals from whom it is not possible to obtain consent, including proxy or advance consent, should be done only if the physical/mental condition that prevents obtaining informed consent is a necessary characteristic of the research population. The specific reasons for involving research subjects with a condition that renders them unable to give informed consent should be stated in the experimental protocol for consideration and approval of the review committee. The protocol should state that consent to remain in the research should be obtained as soon as possible from the individual or a legally authorized surrogate.
18. Both authors and publishers have ethical obligations. In publication of the results of research, the investigators are obliged to preserve the accuracy of the results. Negative as well as positive results should be published or otherwise publicly available. Sources of funding, institutional affiliations and any possible conflicts of interest should be declared in the publication. Reports of experimentation not in accordance with the principles laid down in this Declaration should not be accepted for publication.

C. ADDITIONAL PRINCIPLES FOR MEDICAL RESEARCH COMBINED WITH MEDICAL CARE

1. The physician may combine medical research with medical care, only to the extent that the research is justified by its potential prophylactic, diagnostic or therapeutic value. When medical research is combined with medical care, additional standards apply to protect the patients who are research subjects.
2. The benefits, risks, burdens and effectiveness of a new method should be tested against those of the best current prophylactic, diagnostic, and therapeutic methods. This does not exclude the use of placebo, or no treatment, in studies where no proven prophylactic, diagnostic or therapeutic method exists.
3. At the conclusion of the study, every patient entered into the study should be assured of access to the best proven prophylactic, diagnostic and therapeutic methods identified by the study.
4. The physician should fully inform the patient which aspects of the care are related to the research. The refusal of a patient to participate in a study must never interfere with the patient-physician relationship.
5. In the treatment of a patient, where proven prophylactic, diagnostic and therapeutic methods do not exist or have been ineffective, the physician, with informed consent from the patient, must be free to use unproven or new prophylactic, diagnostic and therapeutic measures, if in the physician's judgment it offers hope of saving life, re-establishing health or alleviating suffering. Where possible, these measures should be made the object of research, designed to evaluate their safety and efficacy. In all cases, new information should be recorded and, where appropriate, published. The other relevant guidelines of this Declaration should be followed.

# Appendix III: MEDWATCH Serious Adverse Event Reporting – Form 3500


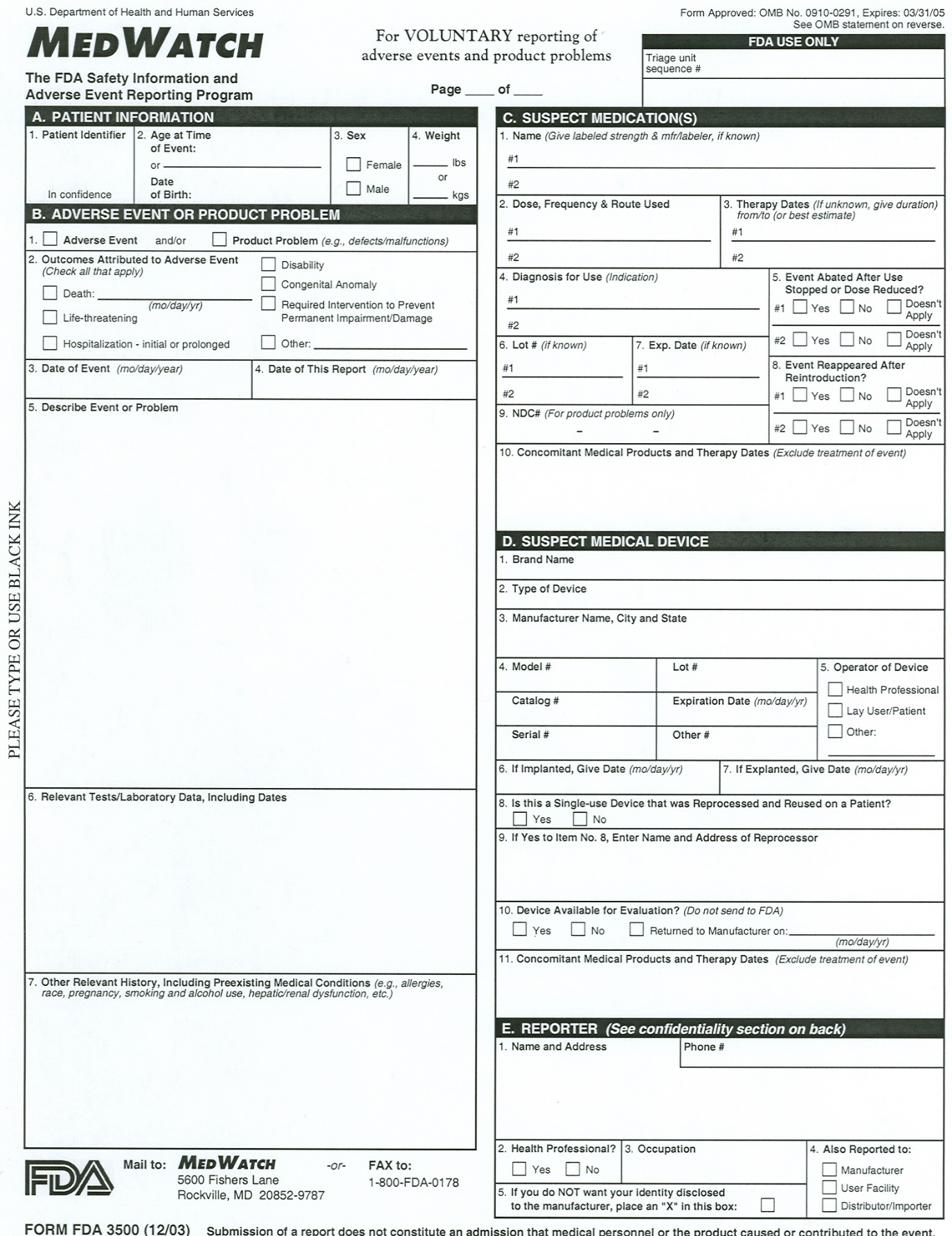


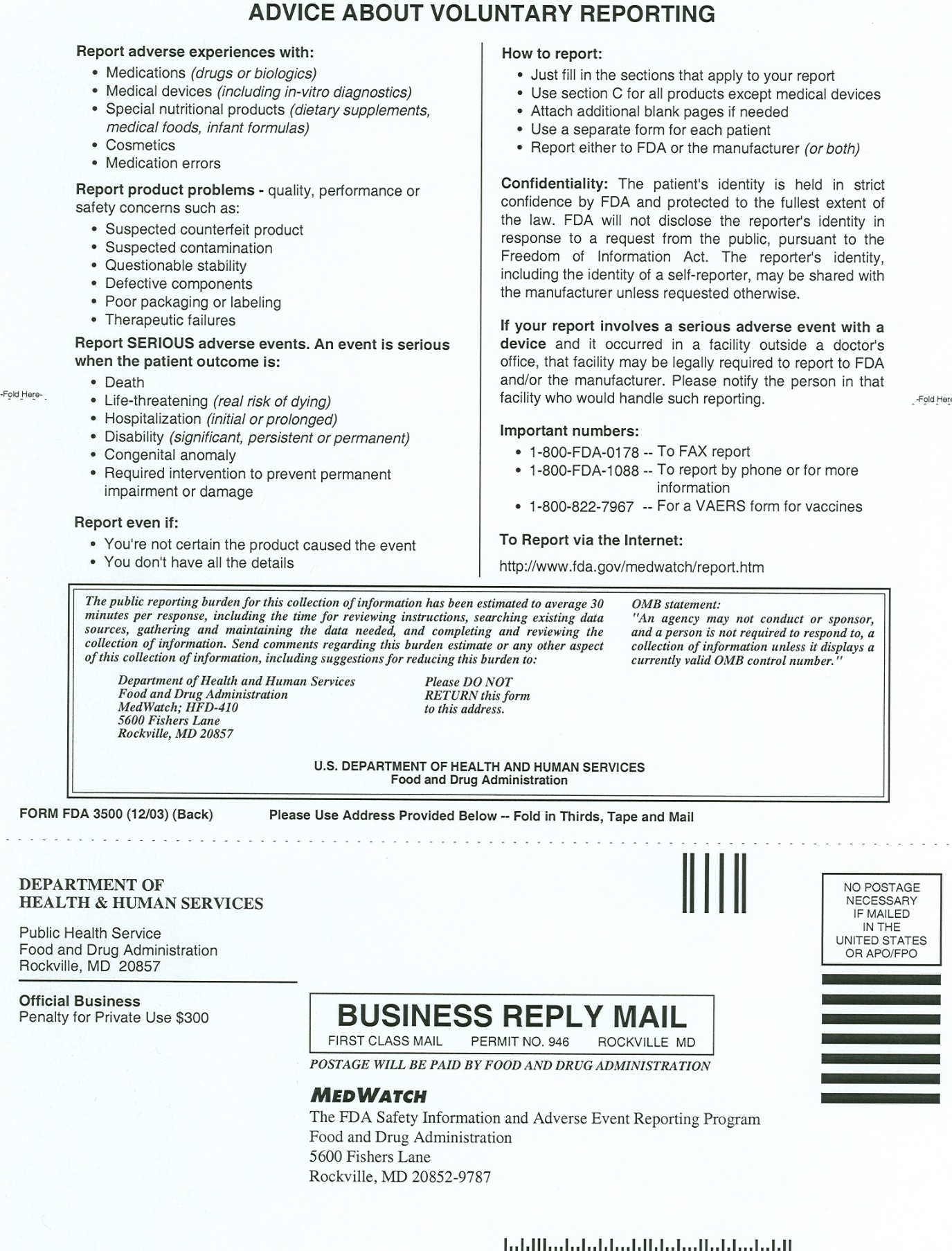


1. Within 60 days prior to Baseline [↑](#footnote-ref-1)
2. Assessments may be done up to 7 days prior to first infusion of Tysabri [↑](#footnote-ref-2)
3. Includes pulse and blood pressure, with additional weight assessment done every 6 months [↑](#footnote-ref-3)
4. Done at investigator’s discretion and may include pregnancy test, if applicable [↑](#footnote-ref-4)
5. Blood draw consists of hematology and serum chemistry. Screening to include hepatitis B and C and HIV testing. Week 24 to include anti-Natalizumab (Tysabri) antibody blood test. [↑](#footnote-ref-5)
6. Done after all other assessments for that visit [↑](#footnote-ref-6)
